# Strategic variations in sarbecovirus and merbecovirus Nsp1 linker regions for translation inhibition

**DOI:** 10.1093/nar/gkag017

**Published:** 2026-01-15

**Authors:** Ruixi Yan, Mingbo Wu, Xiangyu Ge, Qianqian Jin, Moyu Wang, Haolong Zhou, Yan Li, Yue Wang, Shuai Yuan

**Affiliations:** School of Basic Medicine, Tongji Medical College, Huazhong University of Science and Technology, Wuhan, Hubei 430030, China; Hubei Jiangxia Laboratory, Wuhan, Hubei 430200, China; School of Basic Medicine, Tongji Medical College, Huazhong University of Science and Technology, Wuhan, Hubei 430030, China; Hubei Jiangxia Laboratory, Wuhan, Hubei 430200, China; Hubei Jiangxia Laboratory, Wuhan, Hubei 430200, China; Hubei Jiangxia Laboratory, Wuhan, Hubei 430200, China; School of Basic Medicine, Tongji Medical College, Huazhong University of Science and Technology, Wuhan, Hubei 430030, China; School of Basic Medicine, Tongji Medical College, Huazhong University of Science and Technology, Wuhan, Hubei 430030, China; Hubei Jiangxia Laboratory, Wuhan, Hubei 430200, China

## Abstract

Nonstructural protein 1 (Nsp1) is a key virulence factor of coronaviruses, and its stable binding to the 40S ribosomal mRNA entry channel facilitates multiple functions, including suppression of host immune responses and degradation of host mRNA. To understand the structural basis of the conserved protein across viral lineages, we determined the cryo-EM structures of Nsp1–40S complexes of four coronaviruses from wild animals. Our results show that all Nsp1 proteins engage the mRNA entry channel via their *C*-terminal domain (CTD), but do not fully restrict the rotational mobility of the 40S head, which retains ∼5° of movement and repositions the Nsp1 linker region. Comparative analysis revealed distinct patterns in the linker regions connecting the *N*- and CTDs. Sarbecovirus Nsp1 contains a longer linker, whereas the merbecovirus Nsp1 adopts a shorter linker that navigates structural constraints more readily. Functionally, we find that linker length correlates with translation inhibition efficiency, suggesting a structural tuning mechanism. Additionally, variations in linker and helix 1 of the CTD among different lineages may serve as molecular markers for viral classification. Together, our results provide a comparative structural framework for understanding how coronavirus Nsp1 proteins modulate host translation and reflect evolutionary adaptations in ribosome engagement.

## Graphical Abstract

**Graphical Abstract:**
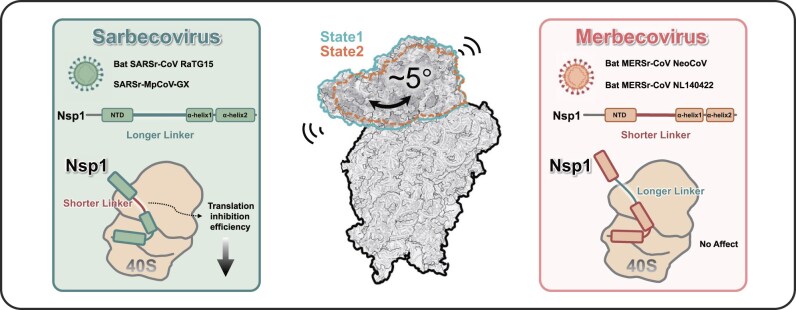

## Introduction

Coronaviruses are enveloped viruses that infect a wide range of hosts, including humans and wild animals. Notably, the sarbecovirus subgenus, which includes severe acute respiratory syndrome coronavirus (SARS-CoV) and severe acute respiratory syndrome coronavirus 2 (SARS-CoV-2), and the merbecovirus subgenus, represented by Middle East respiratory syndrome coronavirus (MERS-CoV), have been responsible for the three pandemics over the past two decades [1–3]. Wild animals such as bats and pangolins serve as natural reservoirs for diverse coronaviruses, posing an ongoing threat for future epidemics. Accordingly, the identification of conserved viral mechanisms between human and animal coronaviruses is critical for developing broad-spectrum antiviral strategies [4–6].

Among the nonstructural proteins (Nsps) encoded by the viral genome, nonstructural protein 1 (Nsp1), which is located at the *N*-terminal of the viral genome, has been proven to suppress the host immune responses in multiple steps and hijack the host translation machineries for the effective production of viral proteins [7–12]. Nsp1 inhibits host protein synthesis, promotes degradation of host mRNAs, and blocks nuclear export of host mRNA by targeting the NXF1–NXT1 mRNA export machinery [7–9, 13–17]. This ∼20-kd protein mainly comprises three domains: a globular *N*-terminal domain (NTD), an alpha helical *C*-terminal domain (CTD), and a flexible linker connecting these two main functional domains. The CTD binds to the 40S ribosomal subunit, effectively blocking the mRNA entry channel and thereby inhibiting host mRNA translation. Detailed high-resolution structures have elucidated the functional mechanisms of the Nsp1 CTD [18–22]. Meanwhile, the NTD interacts with the viral mRNA’s 5′ untranslated region (5′UTR), facilitating the selective translation of viral proteins [23–29]. Furthermore, Nsp1-mediated degradation of host mRNA has been linked to its ribosome binding, underscoring the functional interplay between NTD and CTD [30, 31]. It has been reported that the NTD of Bat-Hp-CoV Nsp1 binds to the decoding center of 40S, revealing that NTD may swap around the 40S subunit head domain [18]. Intriguingly, the linker region between the NTD and CTD of Nsp1 is also pivotal. For instance, a reduction in three residues in the linker region of SARS-CoV-2 Nsp1 decreases viral mRNA translation and potentially leads to reduced virulence [24, 32]. However, the molecular mechanisms of the linker region are still under investigation.

In this study, we investigated Nsp1 proteins from four coronaviruses of wild animal origin, spanning both the sarbecovirus and merbecovirus lineages. We show that all four Nsp1 proteins can suppress protein synthesis in human cells, while the viral 5′UTRs enable escape from translational inhibition. Using cryo-electron microscopy (cryo-EM), we reconstructed two conformational states of the Nsp1-40S complex for each of the four Nsp1 proteins examined. The primary difference between the two states is a rotation of approximately 5° in the 40S ribosomal head domain, suggesting that Nsp1 binding does not fully restrict head movement. This rotation repositions the Nsp1 linker region. Structural comparison revealed lineage-specific strategies: sarbecovirus Nsp1 features a longer linker with limited flexibility, whereas merbecovirus Nsp1 uses a shorter and more flexible linker that accommodates ribosomal constraints. Together with distinct CTD helical patterns, the linker length and flexibility modulate translation inhibition, potentially by influencing tolerance to ribosomal head rotation, which is comparable across both lineages. These findings highlight the structural adaptations in Nsp1 that fine-tune its function in host shutoff and deepen the understanding of the viral lifecycle.

## Materials and methods

### Cloning, expression, and purification of Nsp1 in *E. coli*

Full-length Nsp1 genes of Bat SARSr-CoV RaTG15 (GenBank: OL674077.1), SARSr-MpCoV-GX (GenBank: MT040333.1), Bat MERSr-CoV NeoCoV (GenBank: KC869678.4), and Bat MERSr-CoV NL140422 (GenBank: MG021452.1) were synthesized and cloned into the pET30a(+) vector. Each construct included an *N*-terminal His_6_ tag followed by a TEV protease cleavage site for subsequent purification. In addition, the NTDs of Nsp1 proteins were cloned into the same vector, corresponding to residues 1–133 for SARS-CoV-2, RaTG15, and MpCoV-GX; residues 1–155 for MERS-CoV and NeoCoV; and residues 1–157 for NL140422. Expression of the full-length proteins and NTDs of Nsp1 was performed in Escherichia coli BL21(DE3) cells. Cultures were initially grown in LB medium at 37°C until the OD600 reached about 0.6, followed by the addition of 1mM isopropyl-β-D-thiogalactopyranoside (IPTG) to induce protein expression. The temperature was then lowered to 16°C, and the expression continued for 18 h. Cells were harvested by centrifugation at 6000 g for 15 min, resuspended in lysis buffer (50 mM Tris–HCl, pH 8.0, 500 mM NaCl, 10 mM imidazole, 5% glycerol, 2 mM TCEP), and lysed using a high-pressure cell homogenizer. The lysate was then centrifuged at 35 000 g for 30 min to remove cell debris. The supernatant containing the soluble protein was applied to a Ni-NTA agarose column (Genscript) for affinity chromatography. Both the full-length proteins and NTDs of Nsp1 were eluted with a buffer containing 20 mM Tris–HCl, pH 8.0, 300 mM imidazole, 150 mM NaCl, and 1 mM TCEP. For the Nsp1 NTD samples used in electrophoretic mobility shift assays (EMSA), the His_6_ tags were removed by overnight digestion with HRV-3C protease at 4°C. Further purification was achieved using size-exclusion chromatography (Superdex™ 200 Increase prepacked column, GE Healthcare). The purified protein was concentrated, flash-frozen in liquid nitrogen, and stored at −80°C.

### Preparation of 40S ribosomal subunits

The human 40S ribosomal subunits were purified following established protocols with slight modifications [33, 34]. HEK293F cells were harvested by centrifugation at 1000 g for 10 min at 4°C. Cell pellets were resuspended in lysis buffer consisting of 50 mM HEPES (pH 7.4), 100 mM potassium acetate, 5 mM magnesium acetate, 1 mM DTT, and 0.5% (v/v) NP-40. To ensure complete lysis, cells were subjected to three cycles of flash-freezing and thawing. The lysate was clarified by centrifugation at 10 000 g for 10 min at 4°C, and the resulting supernatant was layered onto a sucrose cushion (50 mM Tris–HCl, pH 8.0, 500 mM potassium acetate, 25 mM magnesium acetate, 1 mM DTT, and 1 M sucrose). Ultracentrifugation was performed at 56 000 g for 2 h using a TY70 Ti rotor (Beckman Coulter). The resulting ribosomal pellet was resuspended in buffer containing 500 mM KCl to dissociate 80S ribosomes into subunits. The sample was passed through a 100 kDa Amicon Ultra centrifugal filter (Millipore) to concentrate and remove dissociated factors. To facilitate subunit separation, 1 mM puromycin was added, and the mixture was incubated sequentially at 37°C for 20 min, room temperature for 20 min, and on ice for 20 min. The sample was then loaded onto a 20–40% (w/v) sucrose gradient in dissociation buffer and ultracentrifuged at 40 000 g at 4°C for 4 h in an SW41 Ti rotor (Beckman Coulter). The 40S fractions were collected, verified by SDS-PAGE, flash-frozen in liquid nitrogen, and stored at −80°C until use.

### Construct design Nsp1 mutations for cellular assays

For cellular assays, various Nsp1 constructs (both wild-type and mutations) were engineered to include an *N*-terminal His_6_-tag and a 3 × Flag-tag, followed by an HRV 3C protease cleavage site. Full-length Nsp1 coding sequences from SARS-CoV-2, RaTG15, MpCoV-GX, MERS-CoV, NeoCoV, and NL140422 were PCR-amplified and cloned into the pRK5 mammalian expression vector using Seamless Assembly Mix (Abclonal, Cat. No. RK21020). Point mutations targeting conserved KH/KF/KY motifs in the CTD were introduced via site-directed mutagenesis using wild-type templates. The following substitutions were generated: SARS-CoV-2 Nsp1, KH164-165AA; RaTG15 Nsp1, KY164-165AA; MpCoV-GX Nsp1, KH164-165AA; MERS-CoV Nsp1, KY181-182AA; NeoCoV Nsp1, KF181-182AA; and NL140422 Nsp1, KY184-185AA.

An overview of the constructions, which include linker length mutations and linker swapping chimeras, is provided in Supplementary Fig. S1. Linker length modifications involved truncations in sarbecovirus Nsp1 proteins and extensions in merbecovirus Nsp1 proteins. For sarbecovirus Nsp1 proteins, residues S142-L145 were deleted, generating SARS-CoV-2 (Nsp1-L^−^), RaTG15 (Nsp1-L^−^), and MpCoV-GX (Nsp1-L^−^). In merbecoviruses, GSGS insertions were introduced to extend the linker regions between R162-D163 in MERS-CoV, R162-N163 in NeoCoV, and D164-D165 in NL140422, resulting in MERS-CoV (Nsp1-L^+^), NeoCoV (Nsp1-L^+^), and NL140422 (Nsp1-L^+^), respectively.

Linker-swapping chimeras were also constructed by replacing the native linker sequence between the NTD and CTD with that from other viral lineages. The MERS-CoV linker (148–167AA) replaced the corresponding regions in SARS-CoV-2 (126–152AA), RaTG15 (126–152AA), and MpCoV-GX (126–152AA), generating SARS-CoV-2 (Nsp1-ML), RaTG15 (Nsp1-ML), and MpCoV-GX (Nsp1-ML), respectively. Conversely, the SARS-CoV-2 linker (126–152AA) was inserted into MERS-CoV (148–167AA), NeoCoV (148–167AA), and NL140422 (150–170AA), yielding MERS-CoV (Nsp1-SL), NeoCoV (Nsp1-SL), and NL140422 (Nsp1-SL).

To assess the translation efficiency of viral 5′UTRs, luciferase reporter plasmids were generated by fusing the Firefly luciferase (Fluc) gene downstream of various viral 5′UTRs. These constructs were cloned into a linearized pCAGGS vector using the Seamless Assembly Mix. The sequences of all 5′UTRs used in this study are provided in Supplementary Table S1.

### Cell culture, plasmid transfection, and Luciferase assay

HeLa and HEK293T cells were maintained in DMEM supplemented with 10% fetal bovine serum (FBS) at 37°C in a humidified incubator with 5% CO₂. Cells were seeded in 24-well plates and grown to approximately 80% confluence prior to transfection. Transfections were carried out using polyethylenimine (PEI; Vazyme, Cat. No. T101) at a DNA:PEI ratio of 1:3 (w/w).

To compare the translation inhibition efficiencies of Nsp1 proteins from sarbecoviruses and merbecoviruses, HeLa cells were co-transfected with 200 ng of Nsp1 expression plasmid and 500 ng of reporter plasmid encoding β-globin 5′UTR-Fluc. The control group received 500 ng of reporter plasmid alone. The same assay was performed in HEK293T cells under identical conditions. Results were consistent with those from HeLa cells.

For dose-response experiments, HeLa cells were co-transfected with increasing amounts of Nsp1 plasmids (Non, 200 ng, 400 ng, or 600 ng) together with 500 ng of β-globin 5′UTR-Fluc reporter plasmid to assess dose-dependent suppression of luciferase expression.

To evaluate the functional impact of linker length modifications on translational repression, cells were co-transfected with 200 ng of Nsp1 constructs per well (wild-type, truncated or extended linker variants [L^−^/L^+^], linker-swapping chimeras [SL/ML], or KH/KY/KF point mutants) along with 500 ng of reporter plasmid. Controls again received only 500 ng of β-globin 5′UTR-Fluc plasmid.

For testing the ability of coronavirus 5′UTRs to escape Nsp1-mediated translation inhibition, six groups of reporter plasmids (500 ng per well) encoding different viral 5′UTRs fused to Fluc were co-transfected with 200 ng per well of the corresponding Nsp1 plasmid. The β-globin 5′UTR-Fluc served as a negative control. For cross-species analyses, combinations of Nsp1 proteins and non-cognate viral 5′UTRs were used following the same transfection strategy.

At 20 h post-transfection, cells were harvested and lysed in 50 µl of lysis buffer (Dual Luciferase Reporter Assay System, Vazyme; Cat. No. DL-101). A volume of 10 µl of cell lysate was transferred to a black 96-well plate and mixed with 50 µl of luciferase substrate solution. Luminescence was measured using a Synergy H1 plate reader (BioTek).

Data are presented as mean ± SD from three independent biological replicates. For statistical analysis, Welch’s Test for unequal variances was performed to assess the significance of luciferase activity. Raw fluorescence intensities were adjusted according to GAPDH. Results were denoted as not significant (ns), or significant as follows: *P < 0.05, **P < 0.01, ***P < 0.001, and not significant (ns, *P* ≥ 0.05).

### Cell culture, plasmid transfection, and flow cytometry

Stable green fluorescent protein (GFP)-expressing HeLa cells were cultured in DMEM supplemented with 10% fetal bovine serum (FBS) at 37 °C in a humidified incubator with 5% CO₂. When cells reached approximately 80% confluence in 24-well plates, they were transfected with 1000 ng of plasmid DNA per well encoding Nsp1 wild-type, KH/KY/KF motif mutants, or other modified constructs. Transfections were performed using PEI (DNA:PEI ratio of 1:3). At 48 h post-transfection, cells were harvested by treatment with 0.25% trypsin for 3 min at 37 °C, and digestion was stopped with serum-containing medium. Cells were pelleted by centrifugation at 500 g for 5 min at 4 °C, washed with pre-cooled PBS to remove residual serum, and resuspended in 200 µL PBS for flow cytometry. Flow cytometry was conducted using a BD LSRFortessa. Instrument calibration was performed with standard microspheres, and FITC channel (FL1) voltages were set based on untransfected control cells. Forward scatter (FSC) thresholds were set to exclude debris, and doublets were eliminated by gating FSC-H versus FSC-A plots. For each sample, 10 000 single-cell events were acquired. Raw data were analyzed with FlowJo software (v10.8). Live cells were gated using FSC-A/SSC-A scatter plots. GFP-positive thresholds were determined from untransfected GFP-stable HeLa cells, while GFP-negative thresholds were established using parental HeLa cells lacking GFP expression. Geometric mean fluorescence intensity (GeoMFI) of GFP-positive populations was calculated for each Nsp1 construct. Relative GFP expression was expressed as a percentage of the vector control.

Statistical analyses were performed using a two-sample equal variance hypothesis T-test. The results are expressed as an average ± standard deviation from three independent experiments. Significance levels were defined as *P < 0.05, **P < 0.01, ***P < 0.001, and not significant (ns, P ≥ 0.05).

### Cell culture, plasmid transfection, and quantitative real-time PCR analysis

HEK293T cells were cultured and transfected as previously described for the luciferase assay. To examine how linker length alterations affect the host mRNA degradation activity of Nsp1 from sarbecoviruses, cells were co-transfected with 200 ng of Nsp1 constructs (wild-type or truncated linker variants) and 500 ng of a β-globin 5′UTR–Fluc plasmid per well. Control cells received 500 ng of reporter plasmid with 200 ng of empty vector. At 20 h post-transfection, total RNA was extracted using RNAiso Plus (Takara, Cat. 108–95-2), and 1 µg was reverse-transcribed into cDNA using HiScript IV RT SuperMix for qPCR (Vazyme, Cat. R423). Quantitative PCR was performed on a CFX96 Touch Real-Time PCR Detection System (Bio-Rad) with ChamQ Universal SYBR qPCR Master Mix (Vazyme, Cat. DL-101) using the following primers: Fluc (forward 5′-GACGCCAAAAACATAAAGAAAGGCC-3′ and reverse 5′-CGAAGTATTCCGCGTACGTGATG-3′); 18S rRNA (forward 5′-CTCAACACGGGAAACCTCAC-3′ and reverse 5′-CGCTCCACCAACTAAGAACG-3′). All qPCR reactions were performed in technical triplicate. Relative mRNA levels were calculated using the 2^(-ΔΔCq) method (Bio-Rad CFX software). Fluc mRNA was normalized to the 18S rRNA reference gene and then expressed relative to the control group.

### Electrophoretic mobility shift assay (EMSA)

Binding interactions between serial dilutions of Nsp1 NTDs and 5′UTR RNAs were analyzed by EMSA. The 5′UTR RNAs were transcribed *in vitro* using the T7 RNA Transcription Kit (Vazyme, Cat. No. TR101) and purified with RNA Clean Beads (Vazyme, Cat. No. N412). The purified RNAs were refolded by sequential incubation at 53°C, 37°C, and 22°C for 5 min each to achieve stable secondary structures. Binding reactions were performed with 2 µM refolded 5′UTR mRNA and varying concentrations of purified Nsp1 NTDs (0, 30, 60, and 120 μM) in binding buffer (10 mM HEPES, pH 7.4, 20 mM NaCl, 0.2 mM TCEP). The mixtures were incubated at 22°C for 30 min to allow complex formation. Samples were then loaded onto a pre-run 6% native polyacrylamide gel (0.5 × TBE) and electrophoresed at 110 V for 2.5 h at 4°C. Gels were stained with Ultra GelRed (Vazyme; Cat. No. GR501) for 30 min, and RNA–protein complexes were visualized under UV transillumination using a gel imaging system.

### Cryo-EM grid preparation and data collection

Nsp1-40S ribosomal complexes (3.5 μl) were applied to glow-discharged Quantifoil Cu R1.2/1.3 grids (Electron Microscopy Sciences). Grids were glow-discharged for 10 s at 15 mA using a plasma cleaner (PELCO easiGlow) immediately before sample application. Vitrification was performed using a FEI Vitrobot Mark IV (Thermo Fisher Scientific) under the following conditions: blotting force 10, blot time 2 s, wait time 20 s, at 4°C, and 100% humidity. Cryo-EM data were acquired on a JEOL CRYO ARM 300 electron microscope operated at 300 kV, equipped with a Gatan K3 direct electron detector. Images were recorded in counting mode at a nominal magnification of 50 000×, corresponding to a physical pixel size of 0.95 Å. The defocus range was set between −1.0 and −2.5 μm. Movies were collected with a total electron exposure of ∼50 e⁻/Å² distributed over 40 frames.

### Cryo-EM data processing

Micrographs obtained for various Nsp1-40S complexes were processed using CryoSPARC v4.7.1 [35]. In total, 3150 micrographs were collected for the RaTG15 Nsp1-40S complex, 3050 for the MpCoV-GX Nsp1-40S complex, 3381 for the NeoCoV Nsp1-40S complex, and 2856 for the NL140422 Nsp1-40S complex. Raw movies were subjected to Patch Motion Correction and Patch CTF Estimation to correct for beam-induced motion and estimate the contrast transfer function, respectively. Particles were initially picked using the Blob picker, followed by particle extraction and multiple rounds of 2D classification to discard poorly defined or contaminant particles. High-quality particles were used to generate *ab initio* 3D models, which enabled heterogeneous refinement to resolve two distinct structural states for each Nsp1–40S complex. For each conformational state, further processing was carried out using Non-Uniform Refinement and CTF Refinement, resulting in high-resolution density maps. These refinements were essential for improving both global resolution and local map quality across the Nsp1-binding interface and ribosomal features.

### Model building and refinement

The human ribosomal complex structure (PDB: 6ZOJ) was used as the initial model in model building [20]. Manual model adjustment and correction were carried out in COOT [36] and refined using phenix.real_space_refine module in PHENIX [37]. Details of the data collection and the parameters involved in the model correction process are given in the Supplementary Table S2. Furthermore, we used ChimeraX for structural analysis and generation of graphics [38].

### In silico analysis of Nsp1–SL1 interaction

Computational modeling of Nsp1–SL1 interactions was performed using the HADDOCK 2.4 web server following established protocols [36]. For protein preparation, the conserved *N*-terminal globular domains were used, corresponding to residues 1–133 for SARS-CoV-2, RaTG15, and MpCoV-GX, and residues 20–155 for MERS-CoV and NeoCoV, and 20–157 for NL140422. The structures of Nsp1 NTDs from RaTG15, MpCoV-GX, MERS-CoV, NeoCoV, and NL140422 were predicted using AlphaFold. The SL1 RNA structures were modeled using the RNAComposer server.

### Sequence clustering and analyzing

Beta-coronavirus sequences were obtained from the InterPro database [39]. To reduce redundancy, sequences were clustered using CD-HIT [40] with a 90% sequence identity and 90% coverage threshold. Profile Hidden Markov Models (HMMs) were constructed using HMMER [41], based on reference Nsp1 sequences from SARS-CoV and MERS-CoV, and used to identify homologous domains across the dataset with an *E*-value cutoff of 1e-3. Subsequently, multiple sequence alignment was performed using Clustal Omega (ClustalO) [42]. A maximum likelihood phylogenetic tree was generated using IQ-TREE 2 [43] with 1500 ultrafast bootstrap replicates to assess branch support. The phylogenetic relationships and domain architectures were visualized using MEGA [44], along with tidygraph [45] and ggraph [46] in R for network-based visualization.

## Results

### Nsp1 of coronaviruses from bat and pangolin inhibits host protein translation in human cells

**Figure 1.**
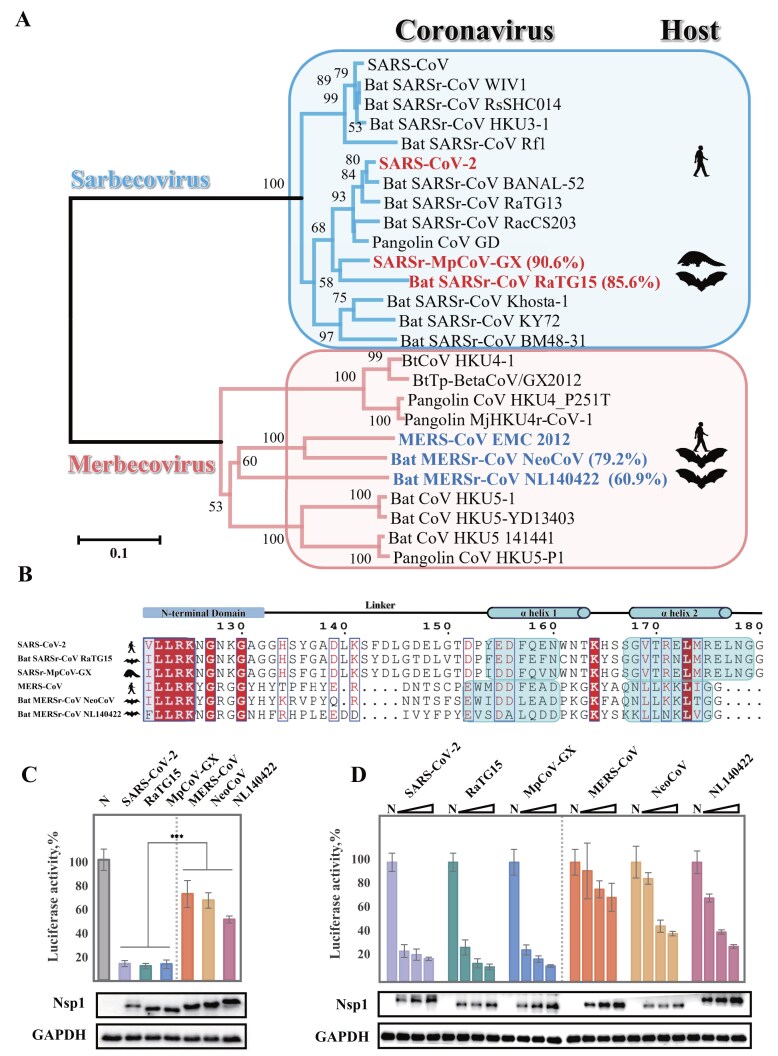
Nsp1 proteins from bat and pangolin coronaviruses inhibit host protein expression in human cells. **(A)** Phylogenetic tree of representative beta-coronaviruses, highlighting the sarbecoviruses (blue box) and merbecoviruses (pink box). Viruses analyzed in this study are shown in bold, with their Nsp1 amino acid sequence identities indicated in parentheses. **(B)** Sequence alignment of Nsp1 proteins from sarbecoviruses and merbecoviruses. Conserved amino acids are highlighted in red. Structural domains are annotated: the *N*-terminal globular domain (blue outline) and two *C*-terminal α-helices (α1 and α2; cyan boxes). A schematic diagram above the alignment shows domain organization. **(C)** Comparative luciferase activity in HeLa cells expressing Nsp1 proteins from sarbecoviruses (SARS-CoV-2, RaTG15, MpCoV-GX; left of dashed line) and merbecoviruses (MERS-CoV, NeoCoV, NL140422; right of dashed line). Nsp1 expression levels were comparable and confirmed by Western blots, with GAPDH as a loading control. Luciferase activity was normalized to the control and adjusted for GAPDH. Data represent mean ± SD from three independent replicates. Statistical significance was assessed by Welch’s Test for unequal variances (***P < 0.001). **(D)** Dose-dependent inhibition of luciferase reporter activity in HeLa cells by Nsp1 proteins. Sarbecovirus Nsp1 proteins (SARS-CoV-2, RaTG15, MpCoV-GX; left of dashed line) and merbecovirus Nsp1 proteins (MERS-CoV, NeoCoV, NL140422; right of dashed line) were expressed at increasing protein levels. Luciferase activity was normalized to the control **(N)** and adjusted for GAPDH expression. Western blots below confirm Nsp1 expression, with GAPDH serving as a loading control. Data are presented as mean ± SD from three independent biological replicates.

In this study, we focused on four Nsp1 proteins of coronaviruses from wild animals to identify universal features of this key viral protein. We selected two sarbecoviruses (bat RaTG15 and pangolin MpCoV-GX) and two merbecoviruses (bat NeoCoV and NL140422). Notably, MpCoV-GX Nsp1 shares 90.6% amino acid similarity with SARS-CoV-2 Nsp1, while RaTG13 Nsp1 exhibits 85.6% similarity. In contrast, the Nsp1 proteins of NeoCoV and NL140422 are more divergent from MERS-CoV Nsp1, with sequence identities of 79.2% and 60.9%, respectively (Fig. 1A). Sequence alignment revealed that the NTD and CTD of Nsp1 proteins are more conserved than the intervening linker regions. Intriguingly, the linker region in merbecovirus Nsp1 is shorter than that in sarbecovirus Nsp1, despite the overall length of merbecovirus Nsp1 being longer (Fig. 1B and Supplementary Fig. S2).

To evaluate the ability of Nsp1 proteins from different beta-coronaviruses to suppress host protein synthesis, we expressed Nsp1 proteins from sarbecoviruses (SARS-CoV-2, RaTG15, MpCoV-GX) and merbecoviruses (MERS-CoV, NeoCoV, NL140422) in HeLa and HEK293T cells, and measured their effects on a luciferase reporter system. Expression of Nsp1 from all six coronaviruses significantly reduced luciferase activity compared to the vector control (Fig. 1C and Supplementary Fig. S3). In Hela cells, among sarbecoviruses, SARS-CoV-2 Nsp1 exhibited a strong inhibition at a relatively lower expression level, reducing reporter activity to 13.54% of the control. In contrast, among merbecoviruses, MERS-CoV Nsp1 showed the weakest suppression, retaining 71.44% of luciferase activity. Other sarbecoviruses (RaTG15 and MpCoV-GX) also strongly inhibited translation (11.56% and 13.12%), while NeoCoV and NL140422 exhibited moderate suppression, retaining 66.18% and 50.27% of activity, respectively.

We next assessed whether the inhibitory effect of Nsp1 proteins on host translation was dose-dependent. Increasing amounts of Nsp1 plasmids led to a progressive reduction in luciferase activity for all tested Nsp1 proteins (Fig. 1D). Sarbecovirus Nsp1 proteins displayed a steep dose-response curve, with substantial inhibition even at lower expression levels. In contrast, merbecovirus Nsp1 proteins showed a more gradual reduction, suggesting differences in their ability to suppress host translation. To further investigate the molecular mechanisms underlying this translation inhibition, we performed structural analysis using cryo-EM reconstruction.

### 40S ribosomal head still rotates with Nsp1 CTD binding in the channel.

**Figure 2.**
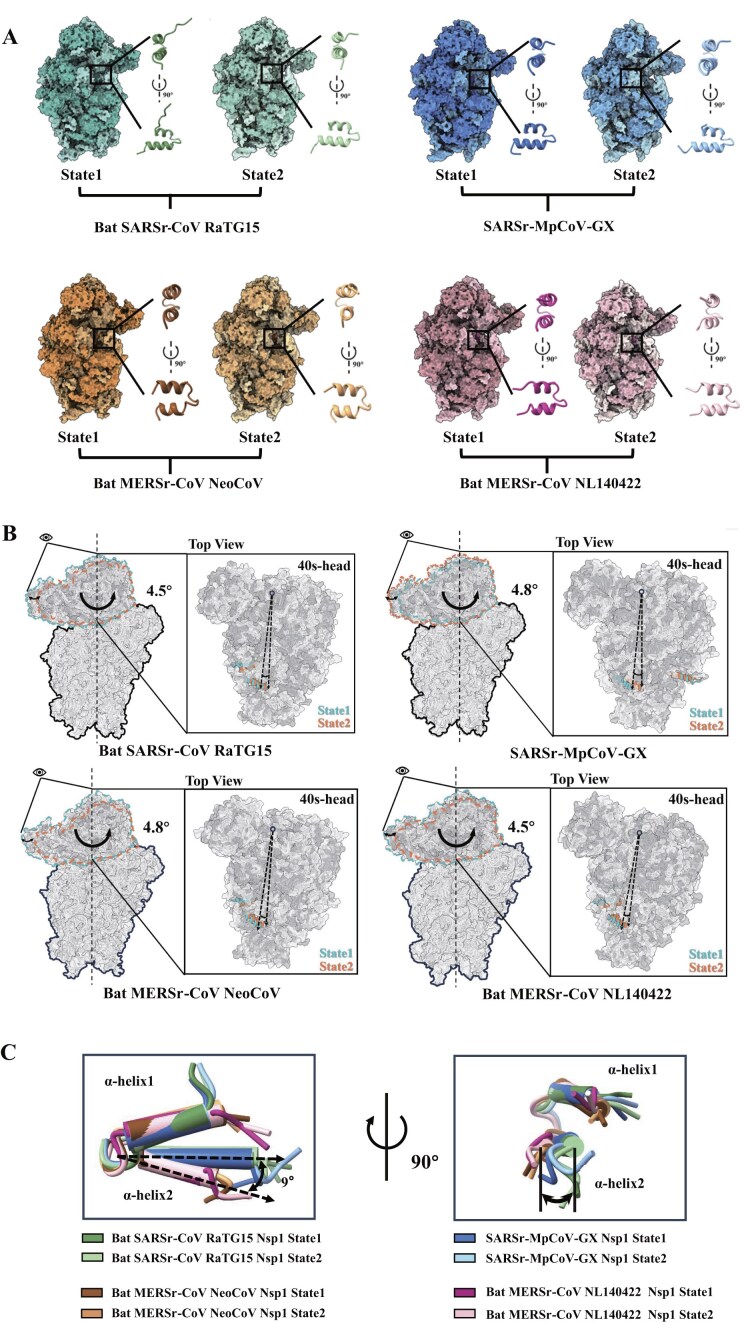
The 40S ribosomal head domain remains flexible with Nsp1 bound in the mRNA channel. **(A)** Two conformational states of Nsp1–40S complexes. The 40S subunit is shown as a surface, and the Nsp1 CTD is highlighted with a box and displayed as a cartoon enlarged on the right, with a 90° rotated view below. States are designated as state 1 (darker) and state 2 (lighter). **(B)** Rotation of the 40S head domain. Structures are aligned on the 40S body, showing the head domain in state 1 (cyan) and state 2 (orange). Insets (right) highlight top views with rotation angles indicated. **(C)** Alignment of Nsp1 CTDs from all eight structures relative to the 40S body. The orientation of helix α2 in sarbecovirus and merbecovirus Nsp1 is labeled to illustrate conformational differences.

Prior studies have demonstrated that coronavirus Nsp1 CTD obstructs the mRNA entry channel in the 40S ribosomal subunit, preventing mRNA loading, and detailed high-resolution structures have been determined [18–22]. In this study, we examined the complex structures of the 40S ribosomal subunit with Nsp1 of the four different coronaviruses mentioned earlier. Surprisingly, we obtained two high-resolution structural models for each Nsp1–40S complex during data processing, which diverge from previous reports and offer new insights into the structural dynamics underlying translation inhibition (Fig. 2A, Supplementary Fig. S4, and Supplementary Table S2). In both observed structures for each Nsp1-40S complex, the CTD of Nsp1 is clearly visible, and the atomic models were constructed (Supplementary Fig. S5). Our comparison of these two conformations revealed that although the CTD of Nsp1 consists of two helices blocking the mRNA entry channel, the 40S subunit head domain still exhibits rotational flexibility. We designated these two conformations as state 1 and state 2 (Fig. 2B, Supplementary Fig. S6 and Supplementary Movies S1–S4). The rotational shift between these states ranges from 4.5° to 4.8°, with no substantial difference observed between complexes containing Nsp1 from sarbecoviruses and merbecoviruses (Fig. 2B).

To further explore this inhibitory mechanism, we examined the conformations of the two helices within each Nsp1 CTD. Interestingly, the positioning of the second helix in the CTD of Nsp1 varies between sarbecoviruses and merbecoviruses. The binding location of helix 2 in RaTG15 Nsp1 CTD is similar to that in MpCoV-GX, whereas helix 2 of the two merbecovirus Nsp1 proteins adopts a different position, deviating by approximately 9° (Fig. 2C). Interestingly, the rotational movement of the 40S head domain does not influence the positioning of helix 2, nor does it affect helix 1 of the CTD. The conformation of CTD helices remains stable in all four Nsp1 proteins, probably stabilized by conserved hydrophobic interactions. (Fig. 2C and Supplementary Fig. S7).

Furthermore, the mRNA latch, formed by ribosomal protein uS3 and h18 and h34 of the 18S rRNA, is known to play a crucial role in translation [47–50]. Our study found that the conformation of the state 2 complexes of Nsp1-40S from these four coronaviruses resemble the “closed” state of the mRNA latch, which regulates mRNA scanning, while the head rotation of the state 1 complexes is akin to the “open” state that is necessary for mRNA loading (Supplementary Fig. S8). Together, these results collectively indicate that while all the four coronaviruses Nsp1 CTD form a stable barrier in the mRNA entry channel, it does not totally prevent rotational movement of the 40S ribosomal head.

### Interactions between Nsp1 CTD and ribosomal mRNA channel

**Figure 3.**
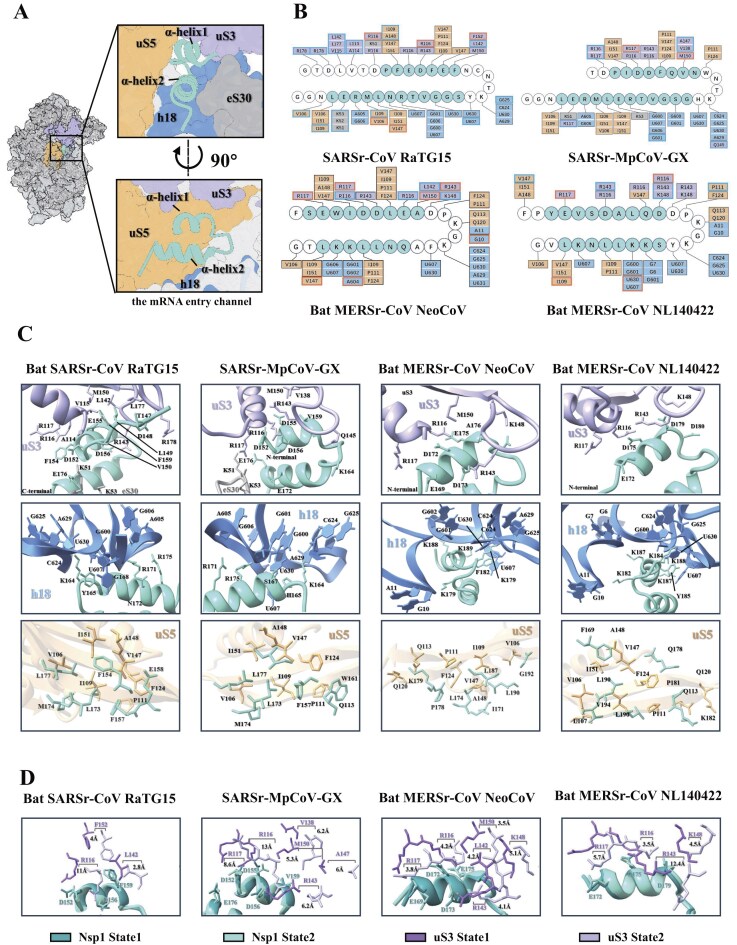
Interactions between the 40S ribosomal subunit and Nsp1 proteins. **(A)** Overall structure of Nsp1 bound to the 40S subunit. The 40S is shown as a surface, and Nsp1 as a cartoon. Close-up and cross-sectional views highlight the Nsp1 CTD (cyan) interactions with ribosomal components: uS5 (yellow), uS3 (light purple), eS30 (light gray), and rRNA helix 18 (h18, cornflower blue). **(B)** Molecular contacts between Nsp1 CTD and 40S subunit. Key residues on Nsp1 α-helices 1 and 2 (lime green text) and their binding partners on the 40S (uS3: light purple; h18: cornflower blue; uS5: yellow) are shown. State-specific interactions are highlighted (bold) using colors (state 1, cyan; state 2, orange), revealing conformational differences. **(C)** Detailed interactions of Nsp1 (lime green) with uS3 (top, light purple), h18 (middle, cornflower blue), and uS5 (bottom, yellow). The sidechains of the key residues are shown and labeled. **(D)** Structural variations in Nsp1–uS3 interactions between states, with changes in residue distances indicated, illustrating conformational rearrangements.

The ribosomal mRNA channel, composed of ribosomal protein uS3, uS5, and h18 of the 18S rRNA, serves as the primary binding site for the Nsp1 CTD (Fig. 3A). Across all the complexes, we consistently observed hydrophobic interactions involving conserved residues between ribosomal protein uS5 and helix 2 of the Nsp1 CTD (Fig. 3B and C). In contrast, interactions with h18 of the 18S rRNA are predominantly electrostatic. Specifically, K164 in sarbecovirus Nsp1 and K181/184 in merbecovirus Nsp1 form direct electrostatic contacts with adjacent rRNA nucleotides, while the neighboring H165/Y165 (sarbecoviruses) or F182/Y185 (merbecoviruses) engage in π–π stacking interactions with rRNA bases. These observations highlight the functional importance of a conserved “KH/KY/KF” motif for CTD anchoring (Fig. 3B and C). Consistently, mutations of this motif to alanine (AA) significantly impair translation inhibition, as discussed later.

Although the interactions between the Nsp1 CTD and uS5 or h18 remain largely unchanged during head rotation, likely because these components reside in the body of the 40S subunit, interactions with uS3 are affected by conformational changes in the head domain (Fig. 3B). In the state 1 complexes, helix 1 of Nsp1 CTD forms conserved interactions with a negatively charged surface patch on uS3 in all four Nsp1 variants, indicating structural conservation across sarbecoviruses and merbecoviruses (Figs. 3B and C). In contrast, state 2 complexes exhibit notable positional shifts in the interacting residues, ranging from 2.8 Å (e.g. L142 in the RaTG15 Nsp1-40S complex) to 12.4 Å (e.g. R143 in the NL140422 Nsp1-40S complex) (Fig. 3D). Together, these data suggest that while the 40S head rotation alters uS3-mediated interactions, it does not dislodge the Nsp1 CTD from the mRNA entry channel.

### Linker patterns affect the translation inhibition efficiency induced by Nsp1

**Figure 4.**
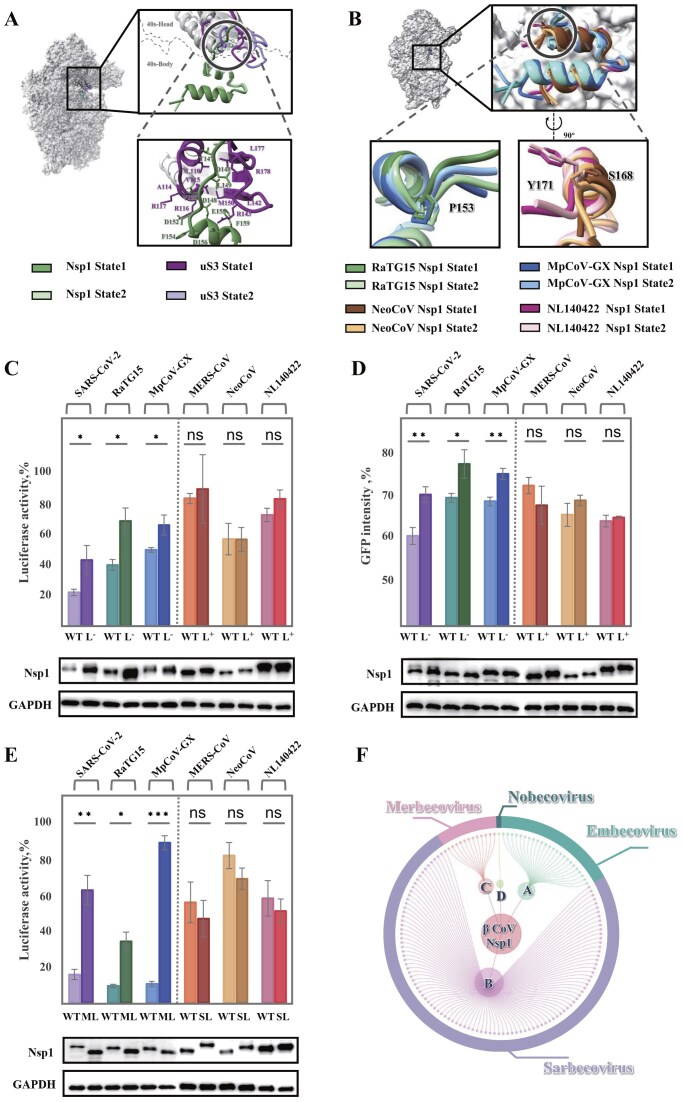
Structural and functional characterization of Nsp1 linker region diversity. **(A)** Cryo-EM structure of the RaTG15 Nsp1–40S complex. The 40S subunit is shown as a surface, and Nsp1 as a cartoon. Insets highlight: (top) conformational changes between state 1 (Nsp1 in dark green, uS3 in dark purple) and state 2 (Nsp1 in light green, uS3 in light purple) relative to uS3; (bottom) enlarged view of detailed interactions between uS3 and the RaTG15 Nsp1 linker in state 1. **(B)** Distinct orientations of Nsp1 linkers in sarbecoviruses versus merbecoviruses. Insets show linker directional patterns with key residues labeled. **(C)** Luciferase reporter assays comparing wild-type (WT) Nsp1 proteins with linker-modified variants. Sarbecoviruses (left) carry shortened linkers (L−), while merbecoviruses (right) carry extended linkers (L+). Reporter activity was measured in HEK293T cells following Nsp1 expression. **(D)** Validation that the linker length of Nsp1 affects translational inhibition efficiency in the stable GFP reporter Hela cell lines. **(E)** Differential translational repression by swapping Nsp1 linkers. Luciferase activity was assessed in HEK293T cells after exchanging the SARS-CoV-2 linker (SL) with merbecoviruses, or the MERS-CoV linker (ML) with sarbecoviruses. **(F)** Classification of β-coronavirus family proteins based on Nsp1 linker regions (inner ring), aligned with classifications derived from conserved regions of full-length genome sequences (outer ring). Western blot confirms Nsp1 expression, with GAPDH as a loading control (C–E). Luciferase activity was normalized to vector control (set as 100%; mean ± SD, *n* = 3) and adjusted for GAPDH levels to account for cellular variations. Statistical significance was determined by Welch’s Test for unequal variances (*** *P* < 0.001, ** *P* < 0.01, * *P* < 0.05, ns *P* ≥ 0.05).

In the RaTG15 Nsp1-40S state 1 complex, we resolved part of the Nsp1 linker region, revealing stabilizing interactions between this segment and ribosomal protein uS3 (Fig. 4A and Supplementary Fig. S9). Specifically, D148 and D152 of RaTG15 Nsp1 form electrostatic interactions with R178 and R116/R117 of uS3, respectively. Additionally, L149 inserts into a hydrophobic pocket on uS3 composed of residues V115, L142, L177, and L182 (Fig. 4A). In contrast, the linker is disordered in the state 2 conformation, suggesting increased flexibility and loss of these interactions in that state (Fig. 4A). Structural comparison of sarbecovirus and merbecovirus Nsp1 proteins reveals notable differences in the linker and adjacent regions. Helix 1 in sarbecovirus Nsp1 is shorter, terminating at a conserved proline residue that redirects the linker, imposing a conformational constraint not presented in merbecovirus Nsp1 (Fig. 4B). The absence of this restriction in merbecoviruses permits the linker to adopt more extended conformations, potentially accommodating ribosomal dynamics more flexibly.

To examine the functional contribution of the linker patterns, we manipulated linker length in both sarbecovirus and merbecovirus Nsp1 proteins. For sarbecoviruses, we truncated the linker (labeled as Nsp1-L⁻); for merbecoviruses, we extended it using a short glycine-serine (GS) insertion (labeled as Nsp1-L⁺) (Supplementary Fig. S1A). Translation inhibition was then assessed. Shortened linkers in sarbecovirus Nsp1 proteins resulted in reduced translation inhibition efficiency, whereas linker extension in merbecovirus Nsp1 proteins did not significantly affect inhibitory efficiency (Fig. 4C). These trends were confirmed using flow cytometry in cells stably expressing GFP, thereby eliminating artifacts from transfection variability (Fig. 4D). The consistency of the results across two different assays supports a genuine linker-dependent mechanism in sarbecovirus Nsp1 proteins. Moreover, we also mutated the KH/KY/KF motif in Nsp1 proteins to alanines (AA). These mutations abolished translational repression both in GFP-expressing stable cells and in luciferase reporter assays (Supplementary Fig. S10). Interestingly, these KH/KY/KF mutations are all in higher expression levels than the wild-type proteins, probably caused by the inability to inhibit protein synthesis. The translation efficiencies of these mutants resemble that of the control group, indicating consistent transfection efficiency and confirming that the observed differences are directly due to Nsp1 activity (Supplementary Fig. S10).

To avoid mutating functional motifs when altering linker length, we also created chimeric Nsp1 constructs by swapping linker regions between sarbecovirus and merbecovirus proteins (Supplementary Fig. S1B). Sarbecovirus Nsp1 proteins carrying the MERS-CoV linker (labeled as Nsp1-ML) showed significantly reduced translation inhibition compared to their wild-type forms. In contrast, merbecovirus Nsp1 chimeras harboring the SARS-CoV-2 linker (labeled as Nsp1-SL) maintained their inhibitory function, suggesting that the inherent flexibility of the merbecovirus linker can tolerate structural substitution (Fig. 4E). Since it’s reported that SARS-CoV-2 Nsp1 mediates host mRNA degradation while MERS-CoV Nsp1 does not [16], we tested whether linker length influences the mRNA degradation efficiency of the sarbecovirus Nsp1 proteins. All three sarbecovirus Nsp1 proteins induced mRNA degradation, whereas shortening the linker reduced this efficiency in cellular assays (Supplementary Fig. S11). Given that the Nsp1 NTD plays a central role in mRNA degradation together with host factor eIF3g, a shorter linker may restrict NTD flexibility, thereby lowering mRNA degradation efficiency. These findings support a model in which the constrained linker of sarbecovirus Nsp1 contributes to the translation inhibition, potentially by better accommodating ribosomal head movements since the ribosomal head repositions the linker region while rotating. This observation may also help explain why a three-residue deletion in the linker of SARS-CoV-2 Nsp1 reduces viral translation and attenuates virulence [24].

Furthermore, we conducted a bioinformatic analysis of Nsp1 sequences from representative beta-coronaviruses, focusing on the region spanning helix 1 and the linker. Distinct, lineage-specific structural patterns were observed across embecoviruses, sarbecoviruses, merbecoviruses, and nobecoviruses (Fig. 4F). Although the current coronavirus taxonomy does not incorporate Nsp1 sequence features, the lineage-specific architecture of the Nsp1 linker may serve as a useful criterion for future classification efforts.

### Viral 5′UTR induces translation inhibition escape

**Figure 5.**
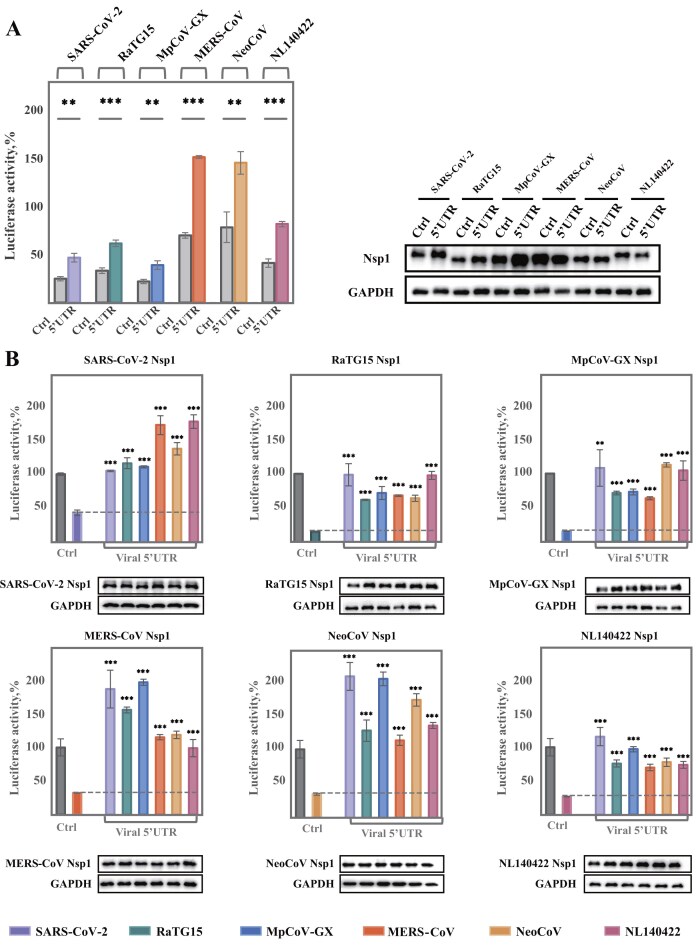
Viral 5′UTR-mediated escape from Nsp1 translational inhibition. **(A)** Relative firefly luciferase activity from reporter constructs containing viral 5′UTRs or control β-globin 5′UTR (Ctrl) in the presence of wild-type Nsp1. Luciferase activity was normalized to control and adjusted for GAPDH (mean ± SD, *n* = 3). Statistical significance was assessed using Welch’s Test for unequal variances (** *P* < 0.01, *** *P* < 0.001). Western blot verified Nsp1 expression across conditions. **(B)** Comparative analysis of 5′UTR-mediated translation escape across coronaviruses. FLuc activity from reporters with different viral 5′UTRs (color-coded) in Nsp1-expressing cells. Controls: no Nsp1 (gray, set as 100%) and no viral 5′UTR (assay control, color-coded). Statistical analysis as in **(A)**. Western blot confirms Nsp1 expression levels. (mean ± SD, *n* = 3).

The 5′ untranslated region (5′UTR) of coronavirus mRNA plays a critical role in evading Nsp1-induced host translation shutoff, enabling selective viral protein synthesis. To assess this function across viral lineages, we tested the 5′UTRs from SARS-CoV-2, MERS-CoV, and the four bat- and pangolin-derived coronaviruses for their ability to promote protein expression in the presence of cognate Nsp1 proteins (Supplementary Table S1). In all cases, the 5′UTR significantly enhanced protein expression despite Nsp1 co-expression, suggesting a conserved mechanism that facilitates viral translation (Fig. 5A). These findings support the model that the 5′UTR collaborates with Nsp1 to suppress host translation while preserving viral mRNA translation, a strategy likely conserved across beta-coronaviruses.

To evaluate whether this 5′UTR-mediated escape is compatible across viral species, and whether it compensates for structural differences in Nsp1 linker regions, we tested each of the six viral 5′UTRs in the presence of the Nsp1 proteins. Remarkably, all six 5′UTRs enabled robust protein expression, indicating that 5′UTR function is probably not strictly lineage-specific (Fig. 5B). Notably, the 5′UTRs of SARS-CoV-2 and MpCoV-GX yield higher expression than the merbecovirus 5′UTRs under the pressure induced by merbecovirus Nsp1 proteins (Fig. 5B). These results suggest that certain 5′UTRs are particularly potent in facilitating translation, even when paired with heterologous Nsp1 proteins. To explore the molecular basis, we performed structural prediction of the Nsp1 NTD and 5′UTR mRNA. Structural comparison of the six Nsp1 proteins revealed a conserved NTD conformation, providing a basis for the observed translation escape (Supplementary Fig. S12). We then examined the binding of Nsp1 NTDs to the six 5′UTRs using EMSA, which confirmed that each Nsp1 NTD can interact with all tested 5′UTRs, consistent with the cellular assays (Supplementary Fig. S13). Despite notable variability in global RNA architecture among the six coronaviruses, the stem loop 1 (SL1) element, a known determinant of Nsp1 resistance, is more conserved (Supplementary Fig. S14). Subsequent *in silico* analyses of Nsp1 NTD–SL1 interactions indicated that all six NTDs may engage the SL1 regions in a conserved binding mode (Supplementary Fig. S15), further providing a structural explanation for the observed cross-species translation escape. This conservation likely underlies the potential cross-species functionality of the 5′UTRs and their ability to mediate escape from Nsp1-induced translation repression.

## Discussion

Coronaviruses have caused three major pandemics in the past two decades and remain a persistent global public health threat. Understanding conserved mechanisms within the viral lifecycle and identifying potential targets for broad-spectrum antivirals are therefore of critical importance. Among the viral proteins, Nsp1—located at the 5′ end of the viral genome in both alpha- and beta-coronaviruses—is notably conserved and plays a pivotal role in antagonizing host immune responses and hijacking the host translation machinery to preferentially synthesize viral proteins [7, 10]. How Nsp1 proteins of different coronaviruses suppress host gene expression is an interesting topic and has been well summarized [8–10, 15, 51, 52]. Especially, the important role of SARS-CoV Nsp1 in suppressing host immune functions and gene expression has been reported in detail [53–62]. MERS-CoV Nsp1 was also found to inhibit host protein synthesis and mediate viral replication by interacting with viral mRNA [63–66]. After the outbreak of the pandemic COVID-19, the properties and molecular mechanisms of SARS-CoV-2 Nsp1 have been revealed [11–13, 23, 24, 26, 27, 67–82]. The host mRNA degradation induced by coronavirus Nsp1 also plays key roles in suppressing host gene expression, and details have been revealed, especially the Nsp1 of SARS-CoV and SARS-CoV-2 [83]. It’s interesting that the host mRNA degradation induced by Nsp1 is independent of the translation inhibition, while the binding of Nsp1 to ribosomes is necessary [16, 30, 31]. Additionally, more Nsp1 proteins, such as those of coronaviruses from human and bat, are also found to be important in viral replication, and a conserved function was suggested [84–90]. What’s more, Nsp1 proteins of alpha-coronavirus viruses share conserved structures with the beta-coronavirus Nsp1 NTD, highlighting the conserved regulation manner of coronavirus in gene regulation [91–98]. Structural investigations of SARS-CoV-2, MERS-CoV, and bat-Hp-CoV Nsp1 proteins have elucidated the molecular basis of ribosomal binding and translation inhibition [18–22]. A recent study shows that SARS-CoV-2 Nsp1 obstructs the mRNA entry channel of bat ribosomal subunit in a conserved manner [99]. Our prior work also highlighted that SARS-CoV-2 Nsp1 is a key viral factor impacting cellular viability and causing significant changes in the host transcriptome soon after its expression [21]. In this study, we extended the structural and functional characterization to four Nsp1 proteins from bat and pangolin coronaviruses. We found that all four Nsp1 proteins could suppress protein expression in human cells, with sarbecovirus Nsp1 proteins displaying stronger translation inhibition than those of merbecoviruses.

We further determined the cryo-EM structures of Nsp1-40S ribosomal complexes for these four coronaviruses and identified two conformational states for each complex. The principal distinction between the two states is a ∼5° rotation of the 40S ribosomal head domain. This rotation occurred in both sarbecovirus and merbecovirus complexes, suggesting that Nsp1 of both sarbecovirus and merbecovirus does not fully restrict ribosomal head domain mobility. Despite this rotation, the Nsp1 CTD remained stably engaged within the mRNA entry channel, indicating a robust mechanism of translation inhibition. Notably, helix 2 of the CTD adopts different positions between sarbecovirus and merbecovirus Nsp1 proteins, revealing lineage-specific structural variations. Intriguingly, the linker is found to bind stably in the state 1 complex instead of the state 2 complex, revealing that the ribosomal head rotation repositions the Nsp1 linker while rotating. This repositioning may also influence the orientation of the NTD, as the linker directly connects to the NTD. It may also help to explain why Nsp1 NTD swaps around the ribosomal head domain. Our analysis highlights notable differences in the linker regions of Nsp1 proteins across coronavirus lineages. In sarbecoviruses, the linker appears structurally constrained in its extension, suggesting that a longer linker is necessary to maintain effective translational inhibition. This is supported by our experimental findings showing that shortening the linker in sarbecovirus Nsp1 significantly reduced its ability to suppress translation. In contrast, extending the linker in merbecovirus Nsp1 had minimal effect on its inhibitory function. These results point to a structural and functional divergence in the evolution of Nsp1 across coronavirus subgenera.

It is worth noting that our structural reconstructions were performed using the human 40S ribosome. However, the core components of the mRNA entry channel—ribosomal proteins uS3 and uS5, along with helix 18 (h18) of the 18S rRNA—are highly conserved among humans, bats, and pangolins (Supplementary Fig. S16). This conservation extends to the critical residues and nucleotides mediating Nsp1 interactions, suggesting that the structural mechanisms we observed are likely applicable across these host species.

During infection, coronaviruses must overcome Nsp1-mediated translational suppression to ensure efficient synthesis of their own proteins. This evasion is mediated by the 5′ untranslated region (5′UTR) of viral mRNAs, which likely facilitates escape by directly interacting with Nsp1 or inducing conformational changes that relieve inhibition. In our study, 5′UTRs from six different coronaviruses were tested, and all were able to restore translation in the presence of Nsp1—even in heterologous Nsp1-5′UTR pairings. This finding suggests a conserved mechanism of Nsp1 evasion among beta-coronaviruses. Supporting this, structural predictions revealed preservation of the binding of Nsp1 NTD and the SL1 element, a key determinant for escaping Nsp1 inhibition. It is noteworthy that the NTD and CTD of Nsp1 are the primary functional domains mediating host translation inhibition and viral RNA escape. The linker region likely contributes indirectly by maintaining the structural flexibility required for the two domains to function properly, rather than by directly influencing 5′UTR-mediated translation rescue.

The NTD of Nsp1 has been implicated in recognizing viral 5′UTRs and facilitating escape from translation inhibition [10, 24, 26, 27, 30, 31, 71, 72]. Nsp1 is also known to promote host mRNA degradation, a process primarily attributed to its NTD [12, 16, 83, 100]. Shortening the linker of sarbecovirus Nsp1 proteins reduces their mRNA degradation efficiency, likely because a shorter linker restricts the flexibility of the NTD. What’s more, the NTD of Bat-Hp-CoV Nsp1 is found to bind to the 40S decoding center and contributes to the translation inhibition [18]. Here in our complexes, NTD is not visible, likely due to conformational flexibility. We propose that repositioning of the linker region may influence the spatial dynamics of the NTD between different ribosome-bound states. Interestingly, in state 2, the linker appears displaced from the ribosome, potentially providing structural insight into how Nsp1 facilitates mRNA decay.

In conclusion, our findings demonstrate that lineage-specific structural features in the Nsp1 linker region, particularly its length and orientation, contribute to differences in host translation inhibition efficiency. These insights offer a deeper understanding of how Nsp1 regulates protein expression and may inform future efforts to design antiviral strategies targeting conserved Nsp1-ribosome interactions.

## Supplementary Material

gkag017_Supplemental_Files

## Data Availability

The structure coordinates and cryo-EM maps from this study are available in the RCSB Protein Data Bank and the Electron Microscopy Data Bank. Accession codes are as follows: 9KMT and EMD-62444 for RaTG15 Nsp1-40S state 1 complex, 9KMU and EMD-62445 for RaTG15 Nsp1-40S state 2 complex, 9KMV and EMD-62446 for MpCoV-GX Nsp1-40S state 1 complex, 9KMW and EMD-62447 for MpCoV-GX Nsp1-40S state 2 complex, 9KMX and EMD-62448 for NeoCoV Nsp1-40S state 1 complex, 9KMY and EMD-62449 for NeoCoV Nsp1-40S state 2 complex, 9KMZ and EMD-62450 for NL140422 Nsp1-40S state 1 complex, 9KN0 and EMD-62451 for NL140422 Nsp1-40S state 2 complex.
