# Supplementary material for: Strategic variations in sarbecovirus and merbecovirus Nsp1 linker regions for translation inhibition
Source: Nucleic Acids Res. 2026 Jan 15;54(2):gkag017. doi: 10.1093/nar/gkag017 (PMC12805902; doi:10.1093/nar/gkag017)
Supplement: gkag017_Supplemental_Files [file gkag017_supplemental_files.zip › NSP1-Supplementary figures and tables-1216.pdf]

Strategic Variations in Sarbecovirus and Merbecovirus Nsp1 Linker Regions for Translation Inhibition

Supplemental information

Supplementary Fig. S1-16

Supplementary Table S1-2

Supplementary Movies. 40S Head Rotation with Nsp1 Binding
Conformational dynamics of the 40S ribosomal subunit upon Nsp1 binding were analyzed using 3D Variability Analysis in CryoSPARC and visualized in UCSF Chimera. The movies illustrate head rotation of the 40S subunit for the following Nsp1-40S complexes:

Movie S1: Bat SARSr-CoV RaTG15 Nsp1-40S complex
Movie S2: SARSr MpCoV-GX Nsp1-40S complex
Movie S3: Bat MERSr-CoV NeoCoV Nsp1-40S complex
Movie S4: Bat MERSr-CoV NL140422 Nsp1-40S complex

Supplementary Fig. S1

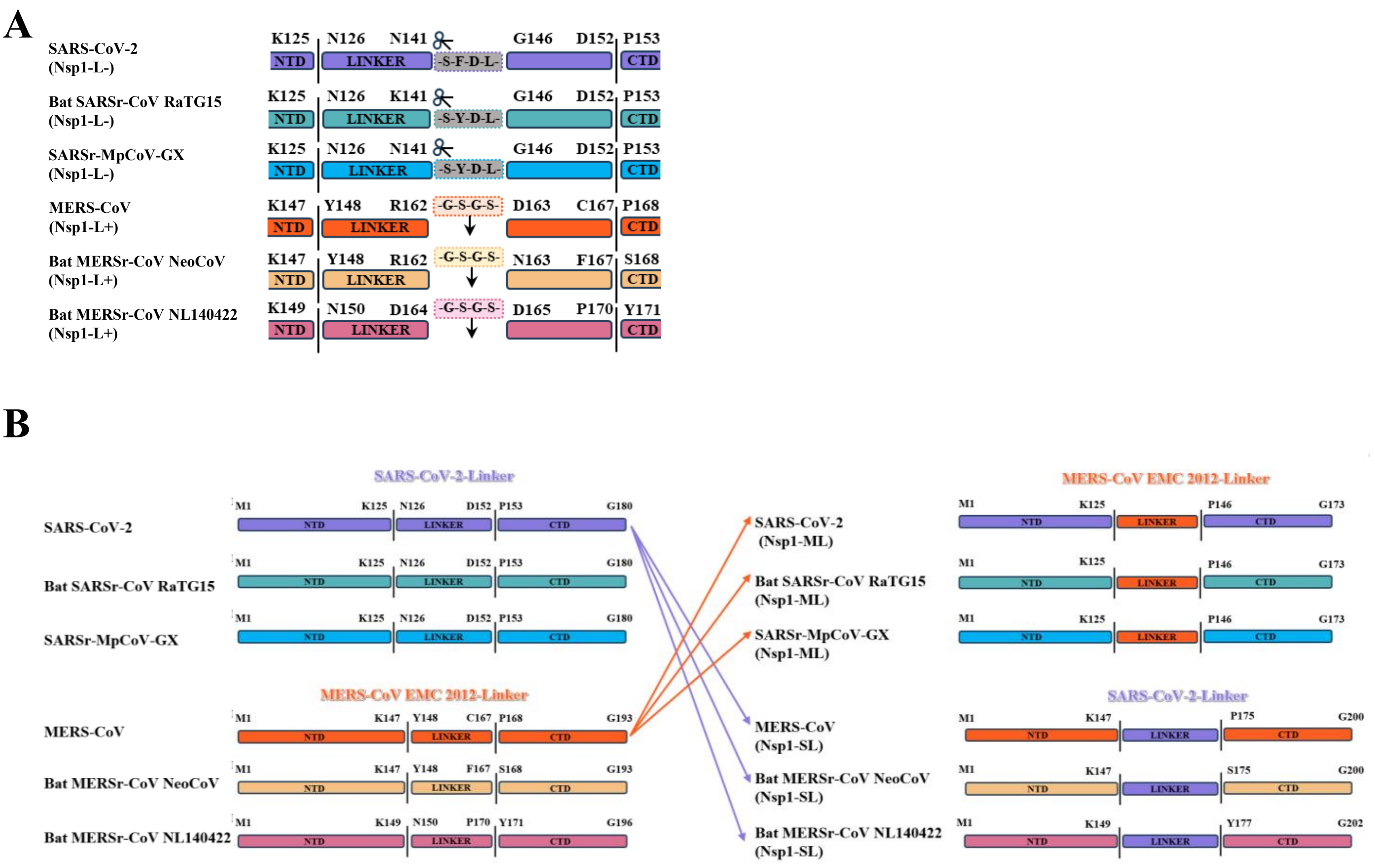

Supplementary Fig. S1. Schematic representation of the Nsp1 mutations with modified linker regions.

(A) Schematic representation of linker length modifications, showing truncation sites and $(GS)_2$ insertion sites within the Nsp1 proteins.

(B) Design strategy for chimeric Nsp1 constructs, illustrating the exchange of linker regions.

Supplementary Fig. S2

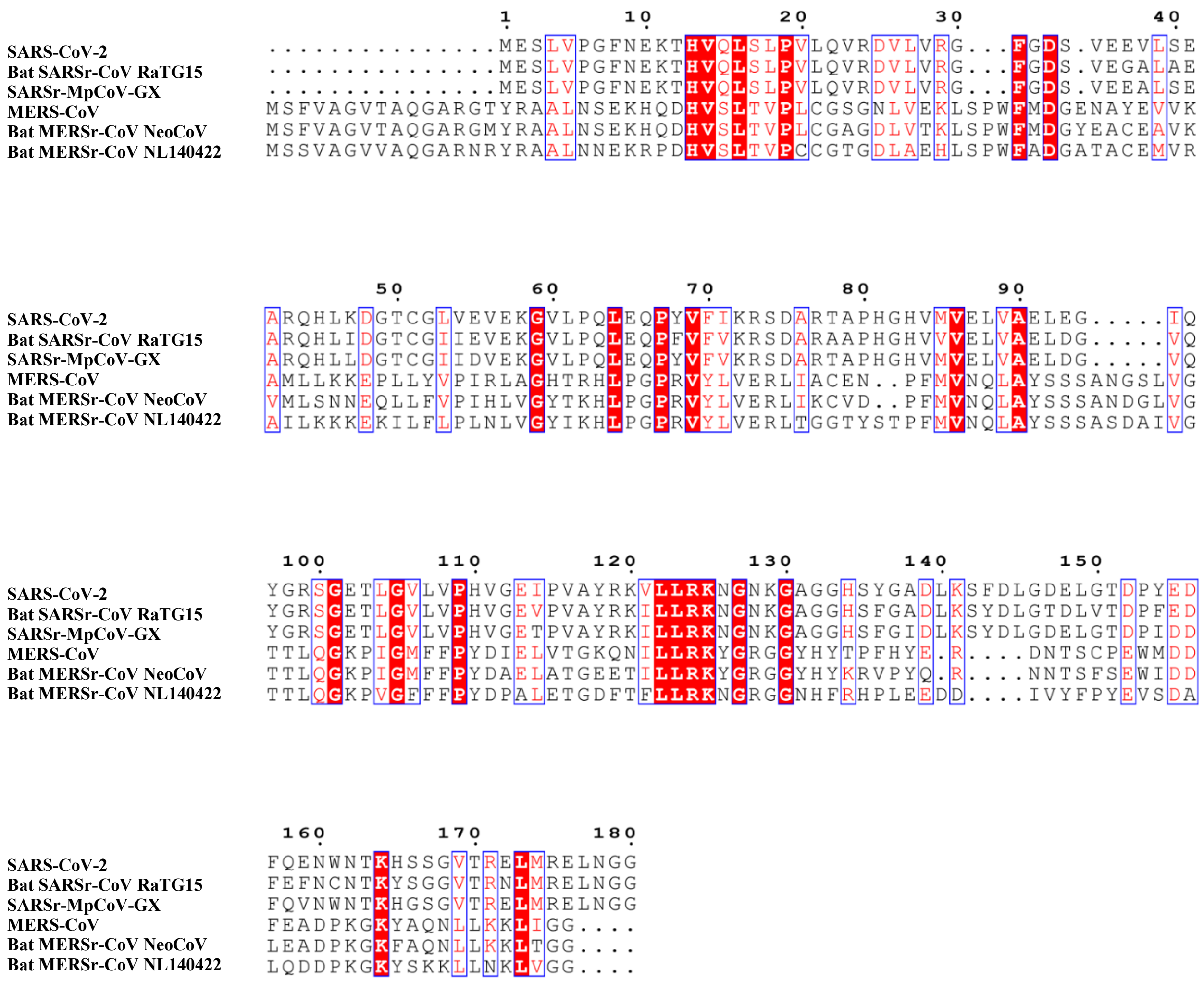

Supplementary Fig. S2. Sequence alignment of full-length Nsp1 proteins.
The full-length amino acid sequence alignment of Nsp1 proteins in this study was performed using CLUSTALW and visualized with ESPript 3.0. Conserved residues are highlighted, with identical residues shown in red boxes and similar residues indicated by red letters.

Supplementary Fig. S3

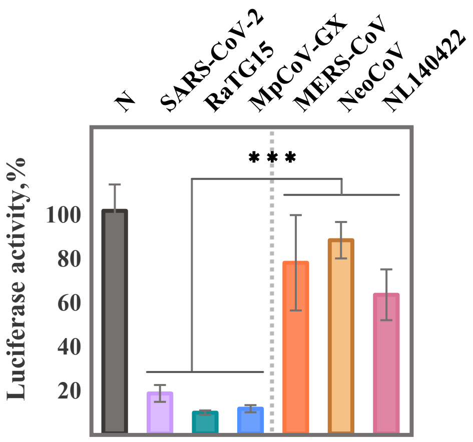

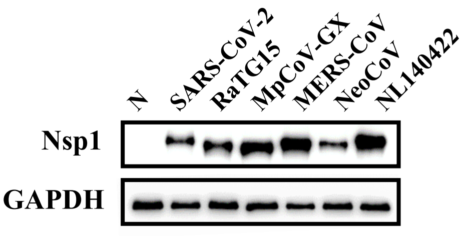

Supplementary Fig. S3. Nsp1 proteins from bat and pangolin coronaviruses inhibit host protein expression in HEK 293T cells.
Comparative luciferase activity in HEK 293T cells expressing Nsp1 proteins from sarbecoviruses (SARS-CoV-2, RaTG15, MpCoV-GX; left of dashed line) and merbecoviruses (MERS-CoV, NeoCoV, NL140422; right of dashed line). Western blot confirms Nsp1 expression, with GAPDH as a loading control. Luciferase activity was normalized to vector control (set as 100%; mean $\pm$ SD, n=3) and adjusted for GAPDH levels to account for cellular variations. Statistical significance was determined by Welch's Test for unequal variances (*** $p < 0.001$).

Supplementary Fig. S4

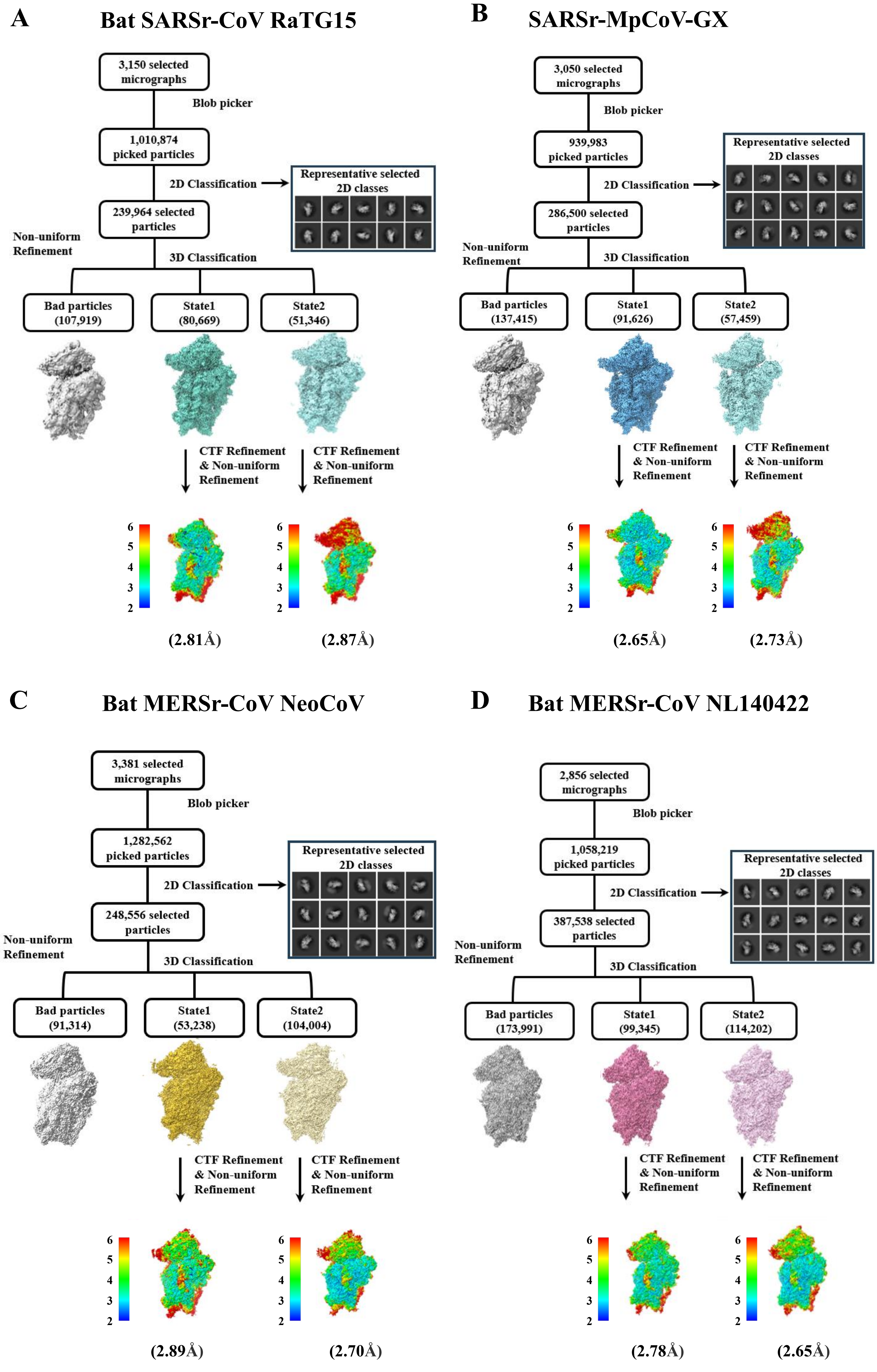

Supplementary Fig. S4. Cryo-EM data processing workflow for Nsp1-40S ribosomal complexes.

Processing pipelines for (A) Bat SARSr-CoV RaTG15 , (B) SARSr-MpCoV-GX, (C) Bat MERSr-CoV NeoCoV, and (D) Bat MERSr-CoV NL140422 Nsp1-bound 40S ribosomal complexes. Representative 2D class averages and corresponding local resolution estimations are displayed for each complex.

Supplementary Fig. S5

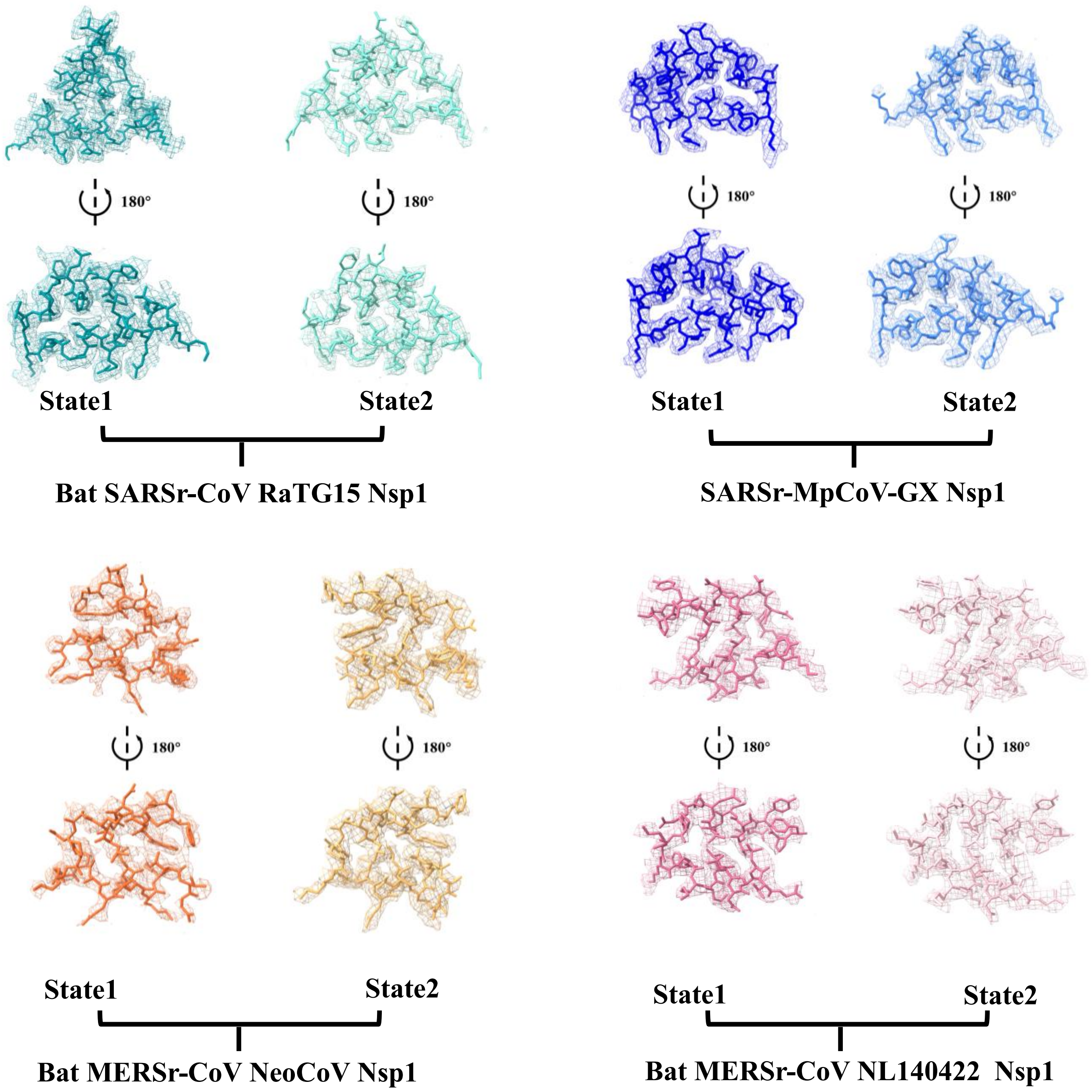

Supplementary Fig. S5. Cryo-EM maps and model-to-map fit the Nsp1 CTD in Nsp1-40S complexes.

Modelled structure with corresponding cryo-EM maps for the Nsp1 CTD are shown as transparent meshes, with State 1 (dark shades) and State 2 (light shades) depicted for each complex. Color coding corresponds to the respective viral strains: Bat SARSr-CoV RaTG15 (cyan), SARSr-MpCoV-GX (blue), Bat MERSr-CoV NeoCoV (yellow), and Bat MERSr-CoV NL140422 (pink).

Supplementary Fig. S6

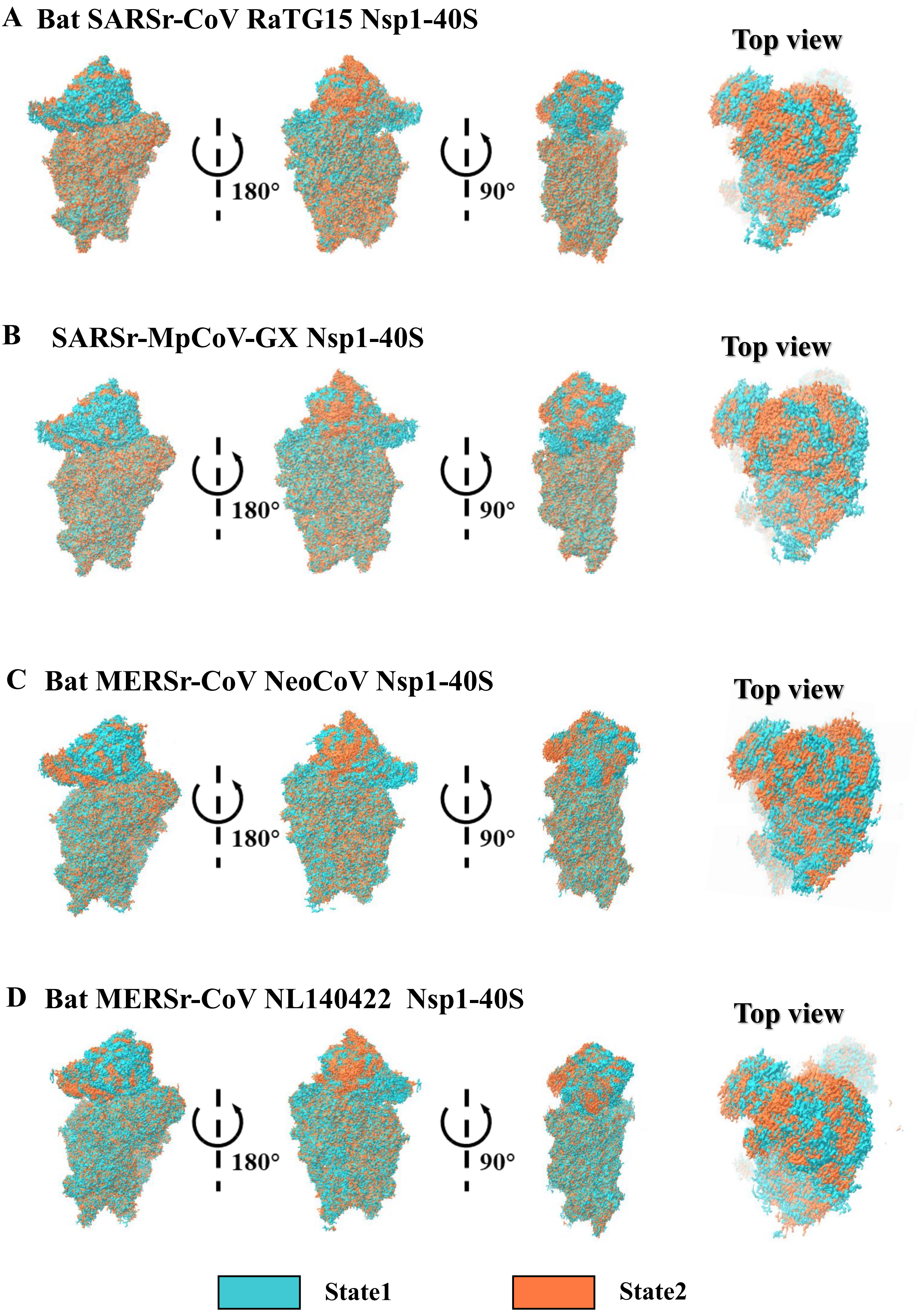

Supplementary Fig. S6. 40S ribosomal head rotation in Nsp1-bound complexes. Two distinct structural states of each Nsp1-40S complex were aligned using the 40S body as a reference. State 1 and State 2 are colored in cyan and orange, respectively. Right panels display top-view of the aligned Cryo-EM maps: (A) Bat SARSr-CoV RaTG15 Nsp1-40S, (B) SARSr-MpCoV-GX Nsp1-40S, (C) Bat MERSr-CoV NeoCoV Nsp1-40S, and (D) Bat MERSr-CoV NL140422 Nsp1-40S complexes.

Supplementary Fig. S7

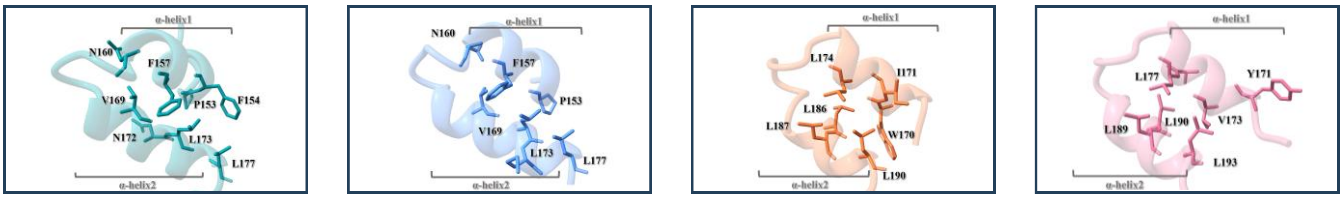

Bat SARSr-CoV RaTG15 **SARSr-MpCoV-GX** **Bat MERSr-CoV NeoCoV** **Bat MERSr-CoV NL140422**

Supplementary Fig. S7. Hydrophobic interactions stabilizing the helices of Nsp1 CTD.

Key residues mediating interhelical hydrophobic interactions within the Nsp1 CTD are explicitly labeled. Structural representations depict the spatial organization of these critical hydrophobic residues that maintain the CTD fold.

Supplementary Fig. S8

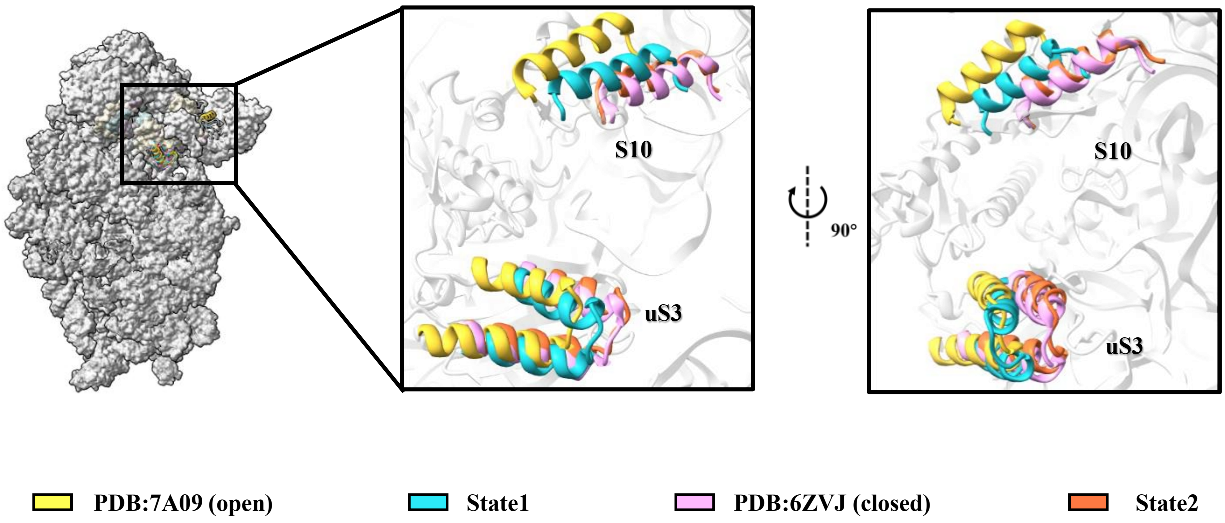

Supplementary Fig. S8. Structural comparison of two states of Nsp1-40S complexes with reference ribosomal structures.

Superposition analysis reveals State 1 of the Nsp1-40S complex (cyan) adopts a conformation similar to the canonical "open" state (bright yellow; PDB: 7A09), while State 2 complex (orange) matches the "closed" state (pink; PDB: 6ZVJ). The magnified view demonstrates the relative spatial arrangements of key helices from ribosomal proteins eS10 and uS3 within the 40S head domain, illustrating the conformational similarities between states.

Supplementary Fig. S9

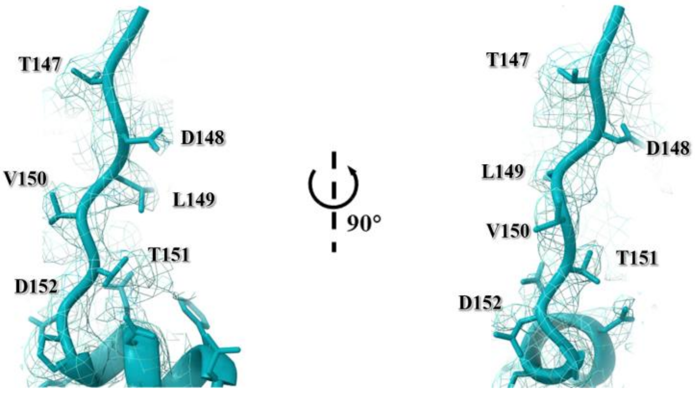

Supplementary Fig. S9. Cryo-EM map and model-to-map fit of the linker region in Bat SARSr-CoV RaTG15 Nsp1 in the State 1 complex.
The reconstructed Nsp1 linker region is displayed with its corresponding cryo-EM map demonstrating model-to-density fit. The residues in the linker region are labeled.

Supplementary Fig. S10

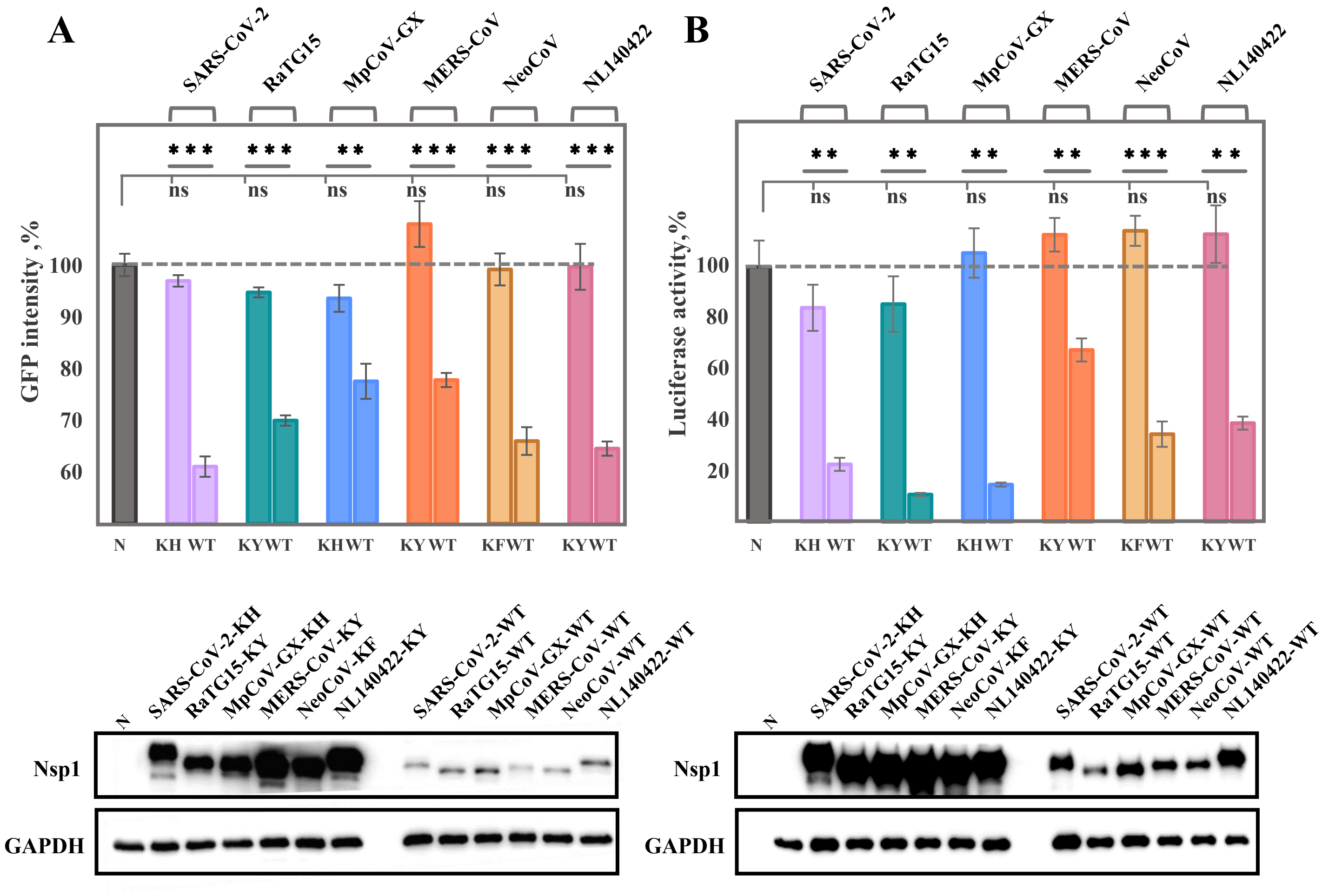

Supplementary Fig. S10. Functional analysis of Nsp1 KH/KY/KF mutations in the translation inhibition.

(A) Loss of translational inhibition by Nsp1 KH/KY/KF-to-AA mutants in stable GFP reporter Hela cells. Relative GFP fluorescence shows abolished inhibition in mutants compared to wild-type (WT). Western blot below shows expression levels of WT and mutant Nsp1 proteins.

(B) Impaired translational repression by Nsp1 KH/KY/KF-to-AA mutants in HEK293T cells. Luciferase reporter assay indicates loss of repression in mutants.

Western blot confirms Nsp1 expression, with GAPDH as a loading control (A-B). Luciferase activity was normalized to vector control (set as 100%; mean ± SD, n=3) and adjusted for GAPDH levels to account for cellular variations. Statistical significance was determined by Welch's Test for unequal variances (*** $p < 0.001$, ** $p < 0.01$, * $p < 0.05$, ns $p > 0.05$).

Supplementary Fig. S11

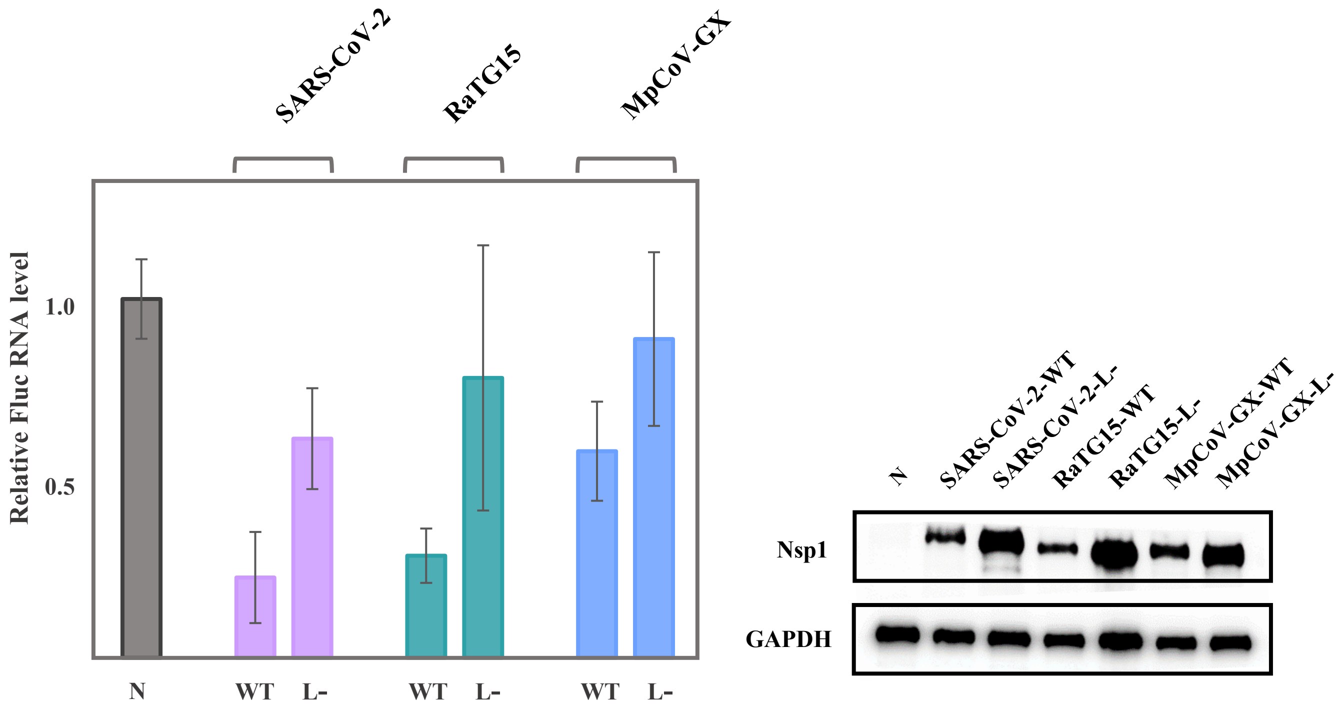

Supplementary Fig. S11. RT-qPCR analysis of mRNA degradation mediated by Nsp1 proteins from sarbecoviruses.

RT-qPCR was used to compare the mRNA degradation activity of wild-type (WT) Nsp1 and shortened linker variants (L−) from SARS-CoV-2, RaTG15, and MpCoV-GX. RNA levels were normalized to the 18S rRNA reference gene and then further normalized to the mean of the control group (N: co-transfected with an equal amount of empty vector). Nsp1 expression was confirmed by Western blot, with GAPDH used as a loading control.

Supplementary Fig. S12

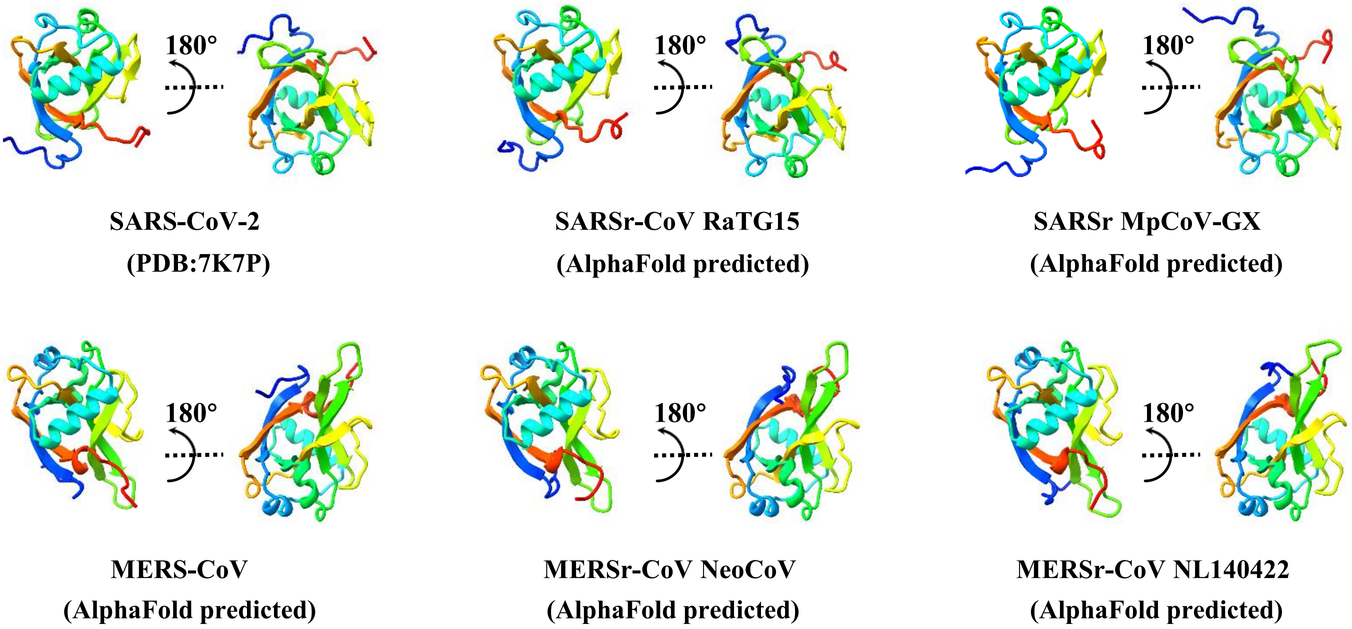

Supplementary Fig. S12. Structural conservation of Nsp1 NTDs.
Structural comparison of the NTDs of Nsp1 from various viral sources, demonstrating a high degree of structural homology. The structures are displayed in cartoon and colored using a rainbow gradient, transitioning from the N-terminus (blue) to the C-terminus (red).

Supplementary Fig. S13

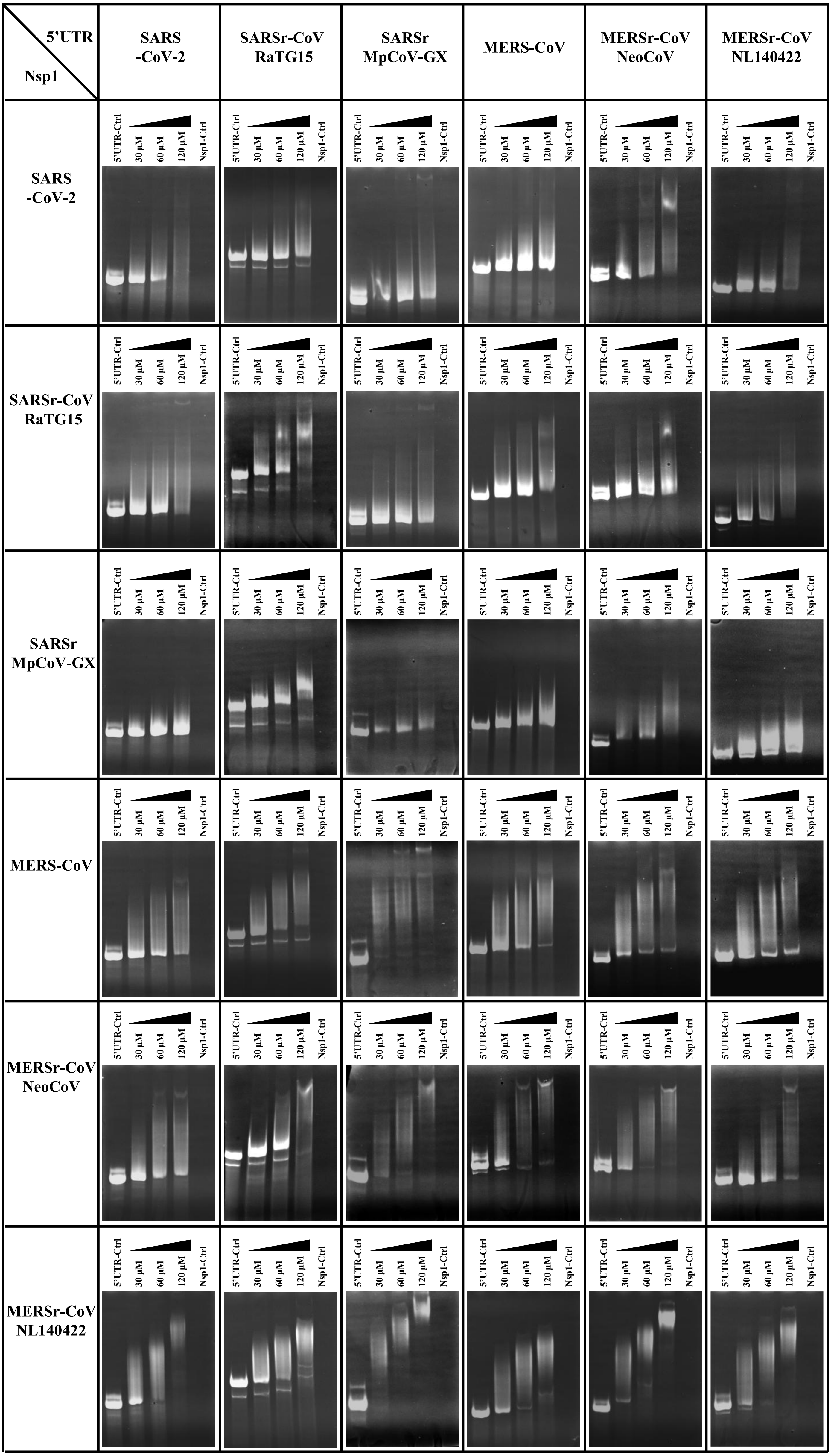

Supplementary Fig. S13. Electrophoretic mobility shift assay (EMSA) showing interactions between Nsp1 NTDs and viral 5'UTRs.

Purified Nsp1 NTDs were incubated with in vitro–transcribed viral 5'UTR mRNAs. The specific Nsp1 NTD and 5'UTR used in each lane are indicated on the left and top of the panel, respectively. The RNA-only control (5'UTR mRNA without protein, 5'UTR-Ctrl) is shown in the leftmost lane, and the protein-only control (120 μM Nsp1 NTD without viral mRNA, Nsp1-Ctrl) is shown in the rightmost lane. In each binding reaction, the concentration of 5'UTR RNA was fixed at 2 μM, while Nsp1 NTD was added at increasing concentrations of 30, 60 and 120 μM. Formations of RNA-protein complexes are indicated by the appearance of slower-migrating bands relative to free RNA. The results show that all tested Nsp1 NTDs can interact with each of the viral 5'UTRs.

Supplementary Fig. S14

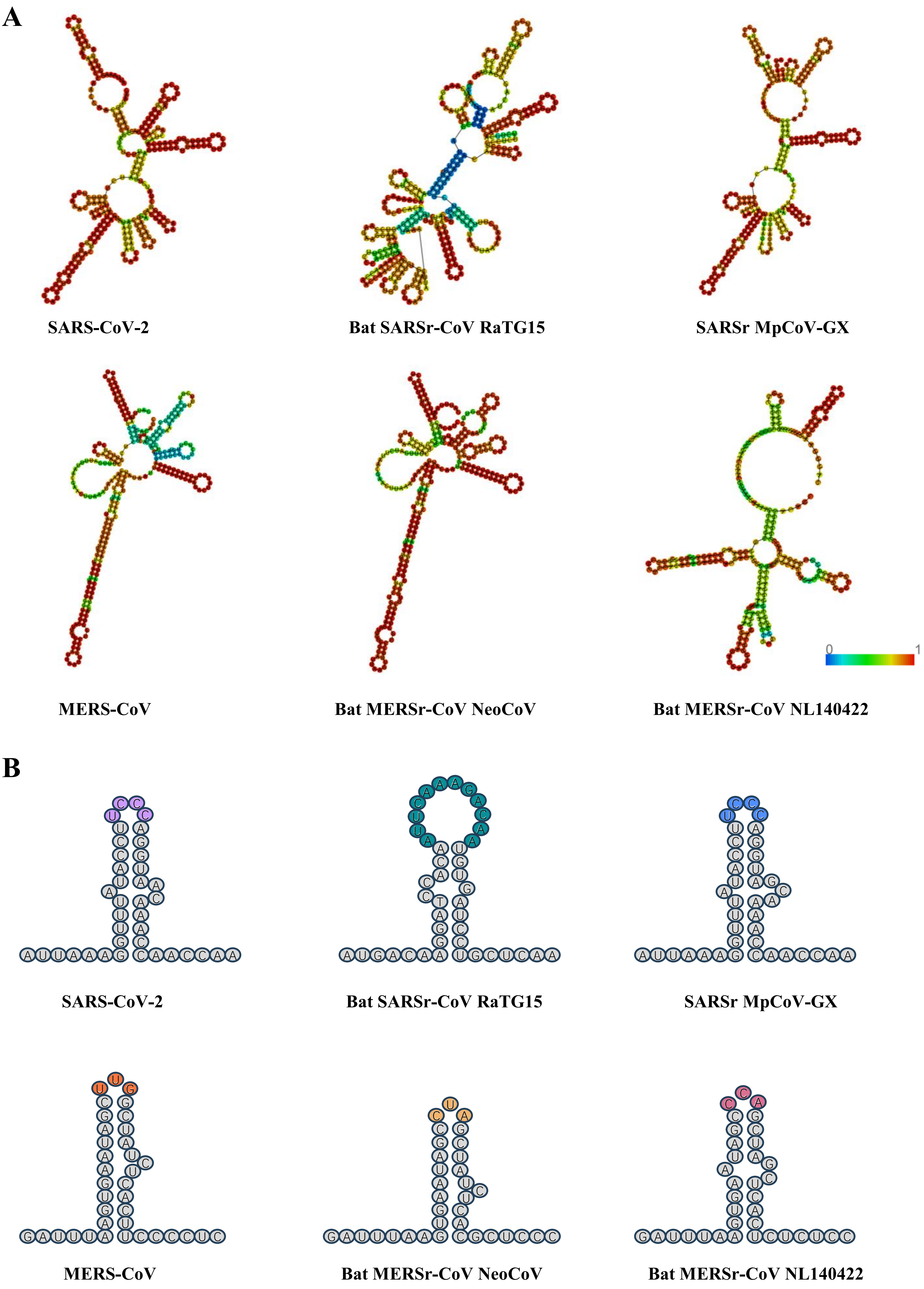

Supplementary Fig. S14. Secondary structure predictions of the 5’UTR and SL1 regions from different coronaviruses.

(A) Predicted secondary structures of the full 5′UTR for SARS-CoV-2, bat SARS-related CoV RaTG15, SARSr MpCoV-GX, MERS-CoV, bat MERS-related CoV NeoCoV, and bat MERS-related CoV NL140422.

(B) Secondary structure models of the stem-loop 1 (SL1) structures from SARS-CoV-2, bat SARS-related CoV RaTG15, SARSr MpCoV-GX, MERS-CoV, bat MERS-related CoV NeoCoV, and bat MERS-related CoV NL140422. The nucleotides within the SL1 loops are highlighted in distinct colors. Predictions were generated using the ViennaRNA Web Services based on minimum free energy (MFE) calculations.

Supplementary Fig. S15

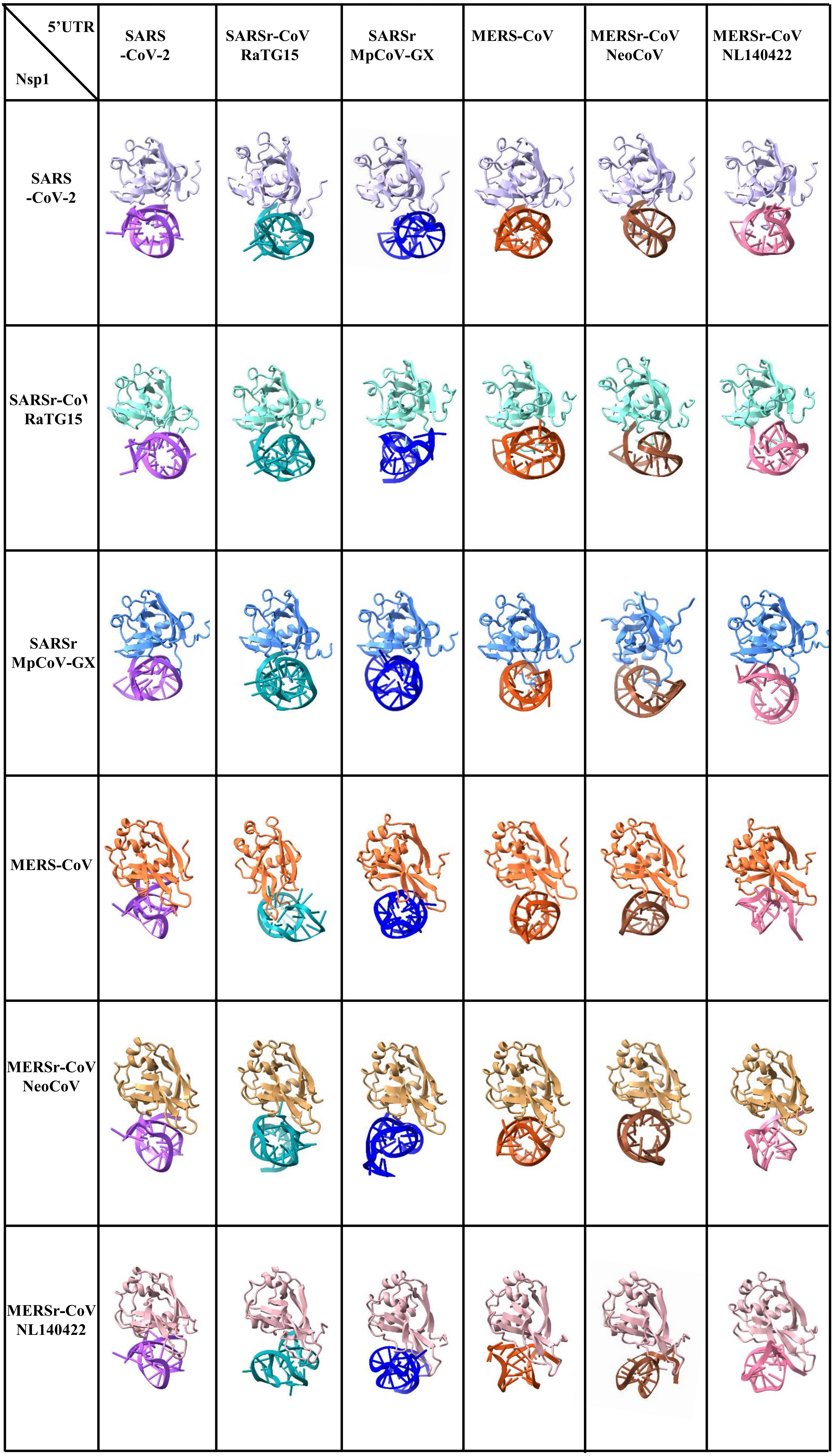

Supplementary Fig. S15. In silico analysis of the interaction between Nsp1 NTDs and SL1 of 5′UTR from six viral species.

The matrix displays the molecular docking results of Nsp1 NTDs with viral SL1s , generated by the HADDOCK web server. It covers both cognate and cross-species interactions, with rows representing SL1 of 5'UTRs and columns representing Nsp1 NTDs.

Supplementary Fig. S16

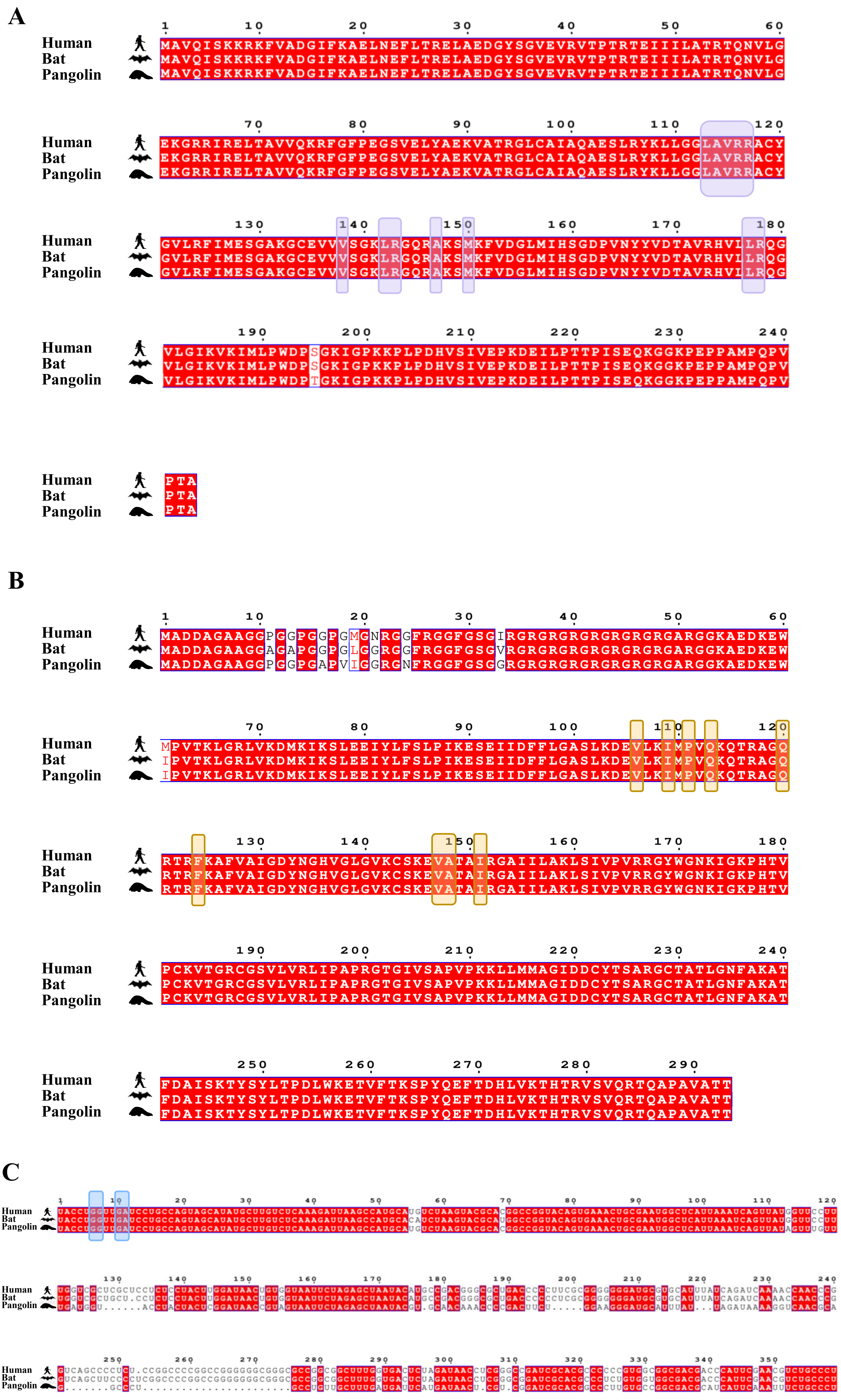

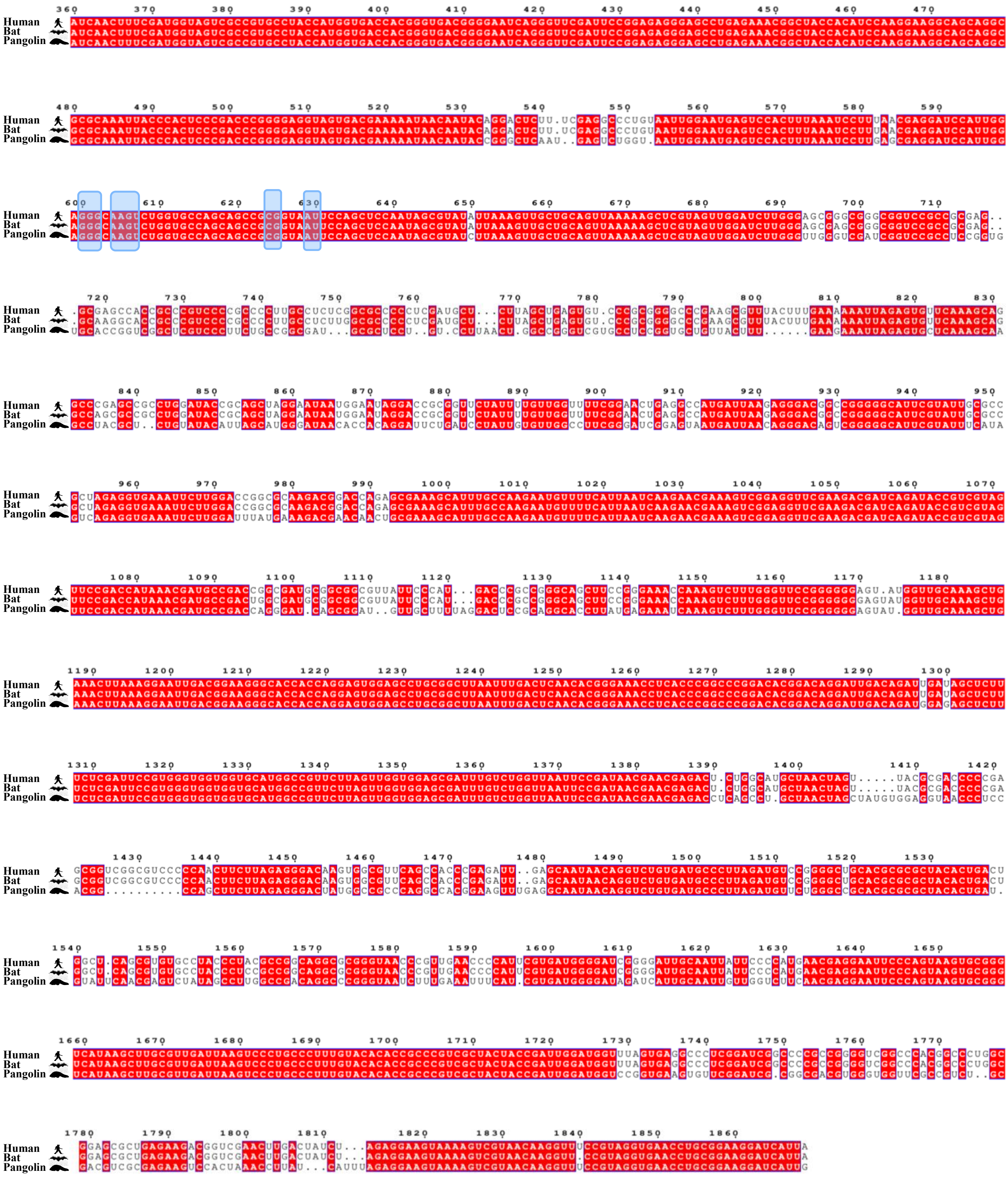

Supplementary Fig. S16. Sequence alignment of the key ribosomal elements from human, bat and pangolin.

(A) uS3, (B) uS5 and (C) 18S rRNA sequence alignments were performed. Conserved residues are highlighted, with identical residues shown in red boxes and similar residues indicated by red letters. The residues or ribonucleotides involved in interactions with Nsp1 are marked with purple boxes (uS3), yellow boxes (uS5) and blue boxes (18S rRNA).

Supplementary Table S1. Sequence of the Viral 5’UTR in this manuscript**.**

Virus	GenBank	Viral 5’UTR Sequences
**SARS-CoV-2**	OU084346.1	AUUAAAGGUUUAUACCUUCCCAGGUAACAAACCAACCAACUUU<br>CGAUCUCUUGUAGAUCUGUUCUCUAAACGAACUUUAAAAUCUG<br>UGUGGCUGUCACUCGGCUGCAUGCUUAGUGCACUCACGCAGUA<br>UAAUUAAUAACUAAUUACUGUCGUUGACAGGACACGAGUAACU<br>CGUCUAUCUUCUGCAGGCUGCUUACGGUUUCGUCCGUGUUGCA<br>GCCGAUCAUCAGCACAUCUAGGUUUCGUCCGGGUGUGACCGAA<br>AGGUAAG
**Bat SARSr-CoV RaTG15**	OL674077.1	AUGACAAGGAUCCACAAUUCAAAGACAAUGUGAUCCUGCUCAA<br>CAAGCACAUUGAUGCUUACAAAACAUUCCCACCCACAGAGUCC<br>AAGAAGGACAAGAUACCUUCCCAGGUAACAAAACCAACCAACC<br>UCGAUCUCUUGUAGAUCUGUUCUCUAAACGAACUUAAAAUCUG<br>UGUUUCUGUCACUGGGCUGUAUGUCUAGUGCACCUACGCAGUA<br>UAAUUAAAAAUCUUUACUGUCGUUGACAGGACACGAGUAACUC<br>GUCCCUCUUCUGCAGAUCACUUACGGUUUCGUCCGUGUUGUGG<br>UCGAUCAUCAGCAUACCUAGGUUUCGUCCGGGUGUGACCGAAA<br>GGUAAG
**SARSr-MpCoV-GX**	MT040333.1	AUUAAAGGUUUAUACCUUCCCAGGUAGCAAAACCAACCAACUC<br>UCGAUCUCUUGUAGAUCUGUUCUCUAAACGAACUUUAAAAUCU<br>GUGUGGCUGUCACUUGGCUGCAUGCCUAGUGCACUCACGCAGU<br>AUAAUAAUAAUUAAUUACUGUCGUUGACAGGAAACGAGUAACU<br>CGUCCGUCUUCUGCAGACUGCUUACGGUUUCGUCCGUGUUGCA<br>GUCGAUCAUCAGCAUACCUAGGUUUUGUCCGGGUGUGACCGAA<br>AGGUAAG
**MERS-CoV**	NC_019843.3	GAUUUAAGUGAAUAGCUUGGCUAUCUCACUUCCCCUCGUUCUC<br>UUGCAGAACUUUGAUUUUAACGAACUUAAAUAAAAGCCCUGUU<br>GUUUAGCGUAUCGUUGCACUUGUCUGGUGGGAUUGUGGCAUUA<br>AUUUGCCUGCUCAUCUAGGCAGUGGACAUAUGCUCAACACUGG<br>GUAUAAUUCUAAUUGAAUACUAUUUUUCAGUUAGAGCGUCGUG<br>UCUCUUGUACGUCUCGGUCACAAUACACGGUUUCGUCCGGUGC<br>GUGGCAAUUCGGGGCACAUC
**Bat MERSr-CoV NeoCoV**	KC869678.4	GAUUUAAGUGAAUAGCCUAGCUAUCUCACGCUCCCCCCGUUCU<br>CUUGAAGAACUUUGAUCUUAACGAACUUAAAUAAAAGCCCUGU<br>UGUUUAACGUAUUGUUGCGCAUUCCUGGUGAGAUUGUGGCAUU<br>AAGUUGCCUGCUCAUCUUGGGUUGUGGACAUAUGUUCAACACU<br>GGGUACAAUUCUAAUUGAAUACUAUUUUUCAGUUAGAGCGUCG<br>UGUCUCUUGUACAUCUCGGUCACAAUAUACGGUUUCGUCCGGU<br>GCGUGGCAAUUCGGGGCACAUC
**Bat MERSr-CoV NL140422**	MG021452.1	GAUUUAAGUGAAUAGCCCAGCUAGCUCACUCUCUCCCGUUCUC<br>UUGCAGAACUUUGAUUUUAACGAACUUAAAUAAAAGCCCUGUU<br>GUAAGGCAUAUUGUUGUGGCUCGCCAGACAUAUGCACUACAUU<br>GGGUACAAUAUUAAUUGAAUACUAUUUUUCAUUUAGAGCGGCG<br>UGUCUCUUGUACCUCUCGGUCACAAUACCCAGUUUCGUCUGGU<br>GCGUGACAAUCCGGGGCACAUC

Supplementary Table S2. Cryo-EM data collection, refinement and validation statistics of 40S-Nsp1 complexes.

**Cryo-EM data collection, refinement and validation statistics of Sarbecovirus Nsp1-40S complexes**				
	**RaTG15-State1 PDB:9KMT EMD-62444**	**RaTG15-State2 PDB:9KMU EMD-62445**	**MpCoV-GX-State1 PDB:9KMV EMD-62446**	**MpCoV-GX-State2 PDB:9KMW EMD-62447**
**Data collection and processing**				
Magnification	50,000×			
Voltage (kV)	300			
Electron exposure (e^-/Å^2)	50			
Defocus size (µm)	-1~ -2.5			
Pixel size (Å)	0.95			
Initial particle images (no.)	1,010,874	1,010,874	939,983	939,983
Final particle images (no.)	80,699	51,346	91,626	57,459
Map resolution (Å)	2.81	2.87	2.65	2.73
FSC threshold	0.143	0.143	0.143	0.143
**Refinement**				
Model resolution (Å)	2.8	2.9	2.9	2.7
FSC threshold	0.143	0.143	0.143	0.143
Map sharpening *B* factor (Å)	45.9	39.4	48.1	41.2
**Model composition**				
Non-hydrogen atoms	74,856	74,598	74,629	74,798
Protein residues	4,905	4,900	4,900	4,900
Nucleotide residues	1,671	1,660	1,662	1,671
***B* factor (Å^2)**				
Protein	82.35	116.17	79.42	99.36
RNA (Nucleotide mean)	77.33	103.68	72.53	89.16
**R.m.s. deviations**				
Bond lengths (Å)	0.003	0.002	0.004	0.006
Bond angles (°)	0.617	0.580	0.656	0.667
**Validation**				
MolProbity score	2.22	2.28	2.35	2.24
Clashscore	7.75	9.33	8.19	8.41
Poor rotamers (%)	3.92	3.43	4.59	3.64
**Ramachandran plot**				
Favored (%)	95.02	94.84	94.08	94.84
Allowed (%)	4.84	4.97	5.78	4.91
Disallowed (%)	0.14	0.19	0.14	0.23

Cryo-EM data collection, refinement and validation statistics of Merbecovirus Nsp1-40S complexes				
	**NeoCoV-State1 PDB:9KMX EMD-62448**	**NeoCoV-State2 PDB:9KMY EMD-62449**	**NL140422-State1 PDB:9KMZ EMD-62450**	**NL140422-State2 PDB:9KN0 EMD-62451**
**Data collection and processing**				
Magnification	50,000×			
Voltage (kV)	300			
Electron exposure ($e^-/Å^2$)	50			
Defocus size (μm)	-1~ -2.5			
Pixel size (Å)	0.95			
Initial particle images (no.)	1,282,562	1,282,562	1,058,219	1,058,219
Final particle images (no.)	53,238	104,004	99,345	114,202
Map resolution (Å)	2.89	2.70	2.78	2.65
FSC threshold	0.143	0.143	0.143	0.143
**Refinement**				
Model resolution (Å)	2.9	2.7	2.8	2.6
FSC threshold	0.143	0.143	0.143	0.143
Map sharpening *B* factor (Å)	41.7	50.8	42.6	48.8
**Model composition**				
Non-hydrogen atoms	73,998	74,538	74,762	74,799
Protein residues	4,896	4,896	4,897	4,897
Nucleotide residues	1,633	1,659	1,669	1,671
***B* factor ($Å^2$)**				
Protein	100.81	66.26	96.22	29.25
Nucleotide residues	90.73	58.36	81.56	27.56
**R.m.s. deviations**				
Bond lengths (Å)	0.003	0.003	0.003	0.004
Bond angles (°)	0.561	0.564	0.588	0.591
**Validation**				
MolProbity score	2.14	2.14	2.31	2.36
Clashscore	7.12	7.56	9.56	12.78
Poor rotamers (%)	3.48	3.46	3.64	3.48
**Ramachandran plot**				
Favored (%)	95.23	95.46	94.53	95.17
Allowed (%)	4.58	4.37	5.32	4.56
Disallowed (%)	0.19	0.17	0.15	0.27
